# Mechanistic Insights into the Adsorption of Monoclonal Antibodies at the Water/Vapor Interface

**DOI:** 10.1021/acs.molpharmaceut.3c00821

**Published:** 2024-01-09

**Authors:** Suman Saurabh, Qinkun Zhang, Zongyi Li, John M. Seddon, Cavan Kalonia, Jian R. Lu, Fernando Bresme

**Affiliations:** † Department of Chemistry, Molecular Sciences Research Hub Imperial College, London W12 0BZ, U.K.; ‡ Biological Physics Group, School of Physics and Astronomy, Faculty of Science and Engineering, the University of Manchester, Manchester M13 9PL, U.K.; § Dosage Form Design and Development, BioPharmaceutical Development, BioPharmaceuticals R&D, AstraZeneca, Gaithersburg, Maryland 20878, United States

**Keywords:** monoclonal antibody, water/vapor interface, protein adsorption, solvent accessible surface area, hydrophobicity, surface activity, surface tension

## Abstract

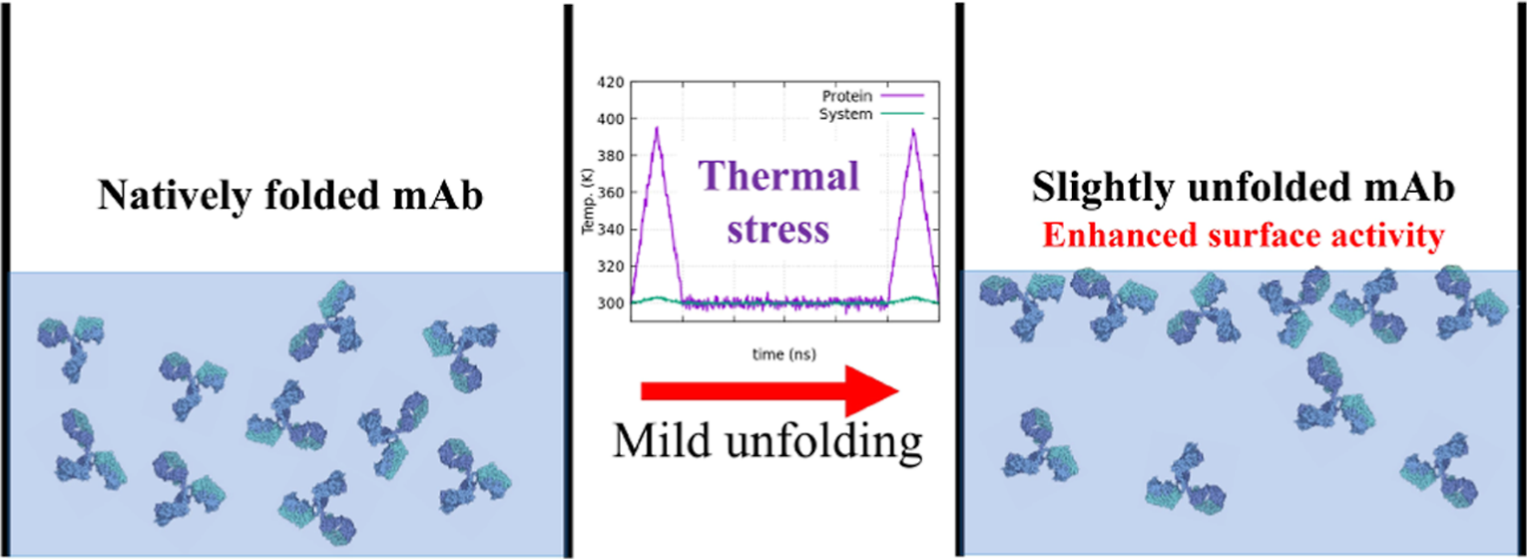

Monoclonal antibodies (mAbs) are active components of therapeutic formulations that interact with the water–vapor interface during manufacturing, storage, and administration. Surface adsorption has been demonstrated to mediate antibody aggregation, which leads to a loss of therapeutic efficacy. Controlling mAb adsorption at interfaces requires a deep understanding of the microscopic processes that lead to adsorption and identification of the protein regions that drive mAb surface activity. Here, we report all-atom molecular dynamics (MD) simulations of the adsorption behavior of a full IgG1-type antibody at the water/vapor interface. We demonstrate that small local changes in the protein structure play a crucial role in promoting adsorption. Also, interfacial adsorption triggers structural changes in the antibody, potentially contributing to the further enhancement of surface activity. Moreover, we identify key amino acid sequences that determine the adsorption of antibodies at the water–air interface and outline strategies to control the surface activity of these important therapeutic proteins.

## Introduction

Monoclonal antibodies (mAbs) are important therapeutic proteins that attract significant attention in cancer and infectious diseases^1−4^ and are used in preoperative diagnostics^5−7^ and prophylaxis.^8−11^ High-concentration (>100 mg/mL) formulations are often required for subcutaneous and intramuscular administration. Developing high-concentration antibody formulations may be challenging because of protein aggregation and particle formation.^12,13^ These critical quality attributes must be controlled to ensure product safety and efficacy.^14−16^ While native proteins might feature aggregation, there is evidence that partial protein unfolding^17^ and mechanical stress^18^ contribute to the aggregation process.

The storage of antibodies in vials and their transportation inevitably involve the interaction of the proteins with interfaces, such as the water/vapor interface, or direct interaction with polymeric, metallic, or mineral surfaces.^19−22^ These interactions result in antibody trapping at the interface, with a potential modification of the protein structure. Such a structural modification may lead to additional aggregation in the bulk solution.

Several experimental studies have demonstrated that antibodies are surface active. Koepf et al.,^23^ measured the adsorption of human immunoglobulin G using surface pressure, IR spectroscopy measurements, Brewster angle, and AFM.^23^ The time-dependent surface pressure isotherms featured a long transient behavior spanning several hours before reaching a plateau. Although adsorption likely starts immediately upon the addition of the antibody to the solution, the experiments highlight the existence of a large dynamic time range for the formation of stationary adsorbed layers, which eventually acquire high viscosity. Long equilibration times in adsorption (probed by measuring surface pressure) were reported in other experiments using different IgG-based antibodies,^24−29^ but we note that the long transient adsorption process is observed in simpler proteins too, such as ovalbumin.^30^

The in situ investigation of proteins at interfaces promoted the use of reflectivity techniques, using either neutrons^25,26,31^ or X-rays^29^ as probes. These experiments provide clear evidence of antibody adsorption at the water/vapor interface and insight into antibody orientation at the interface. The adsorption proceeds through different stages, starting with an induction process with a low interfacial protein concentration, which builds up gradually over time. While there is evidence for the existence of robust β-sheet structure^23,29^ for protein regions adsorbed at the interface, implying that the interfacial protein regions may retain their structure, experiments using environmentally sensitive fluorophores^24^ indicate that the hydrophobicity of protein regions adsorbed at the interface varies with time, suggesting local changes in the protein structure triggered by the interaction of the protein with the interface. Such local structural changes would lead to aggregation of the proteins at the interface and in bulk upon desorption from the interface. Experimental studies have used compression-dilation of the interface to show that the formation and elimination of the interface lead to significant protein aggregation.^32,33^ Tronin et al.^34^ found evidence of an increase in the molecular cross-sectional area of mAbs placed at the water/vapor interface as a function of the exposure time of the mAbs to the interface at low surface pressure, suggesting partial denaturation of adsorbed IgG molecules. The denatured state was necessary to stabilize the mAbs at the interface. Therefore, local unfolding could contribute to antibody adsorption at the water/vapor interface. Further support for unfolding processes can be found in dynamic surface tension measurements and the long-time decay of the surface tension.^29^ The microscopic mechanism determining such an unfolding process and its role in driving aggregation are poorly understood. Indeed, experimental studies using IRRAS indicated that the structure of the adsorbed IgG remained intact with no signature of any structural change.^23^

Understanding the relationship between structural modifications and protein adsorption is of great interest to academia and industry. Computational efforts would help evaluate minor adsorption-induced structural changes in atomic detail. However, simulation force fields for proteins are mostly parametrized with reference to the protein behavior in bulk. In light of this, it is important to evaluate the performance of various MD force fields with respect to their ability to reproduce the experimentally observed adsorption behavior of mAbs. Understanding the differences in the adsorption behavior of antibodies using different force fields would help us to better understand the interplay and underlying importance of various energetic contributors to adsorption. Engin et al. used MD simulation to study the energetics of the adsorption of amphiphilic peptides at the water/vapor interface. Their analysis revealed the dehydration of hydrophobic side chains and surface tension as the major driving forces for adsorption, while loss of orientational entropy upon adsorption at the interface was recognized as the major component promoting desorption. While many other MD studies of peptides and single-domain proteins at the water/vapor interface have been performed,^35−37^ these molecules are far simpler than multidomain proteins like mAbs. Hence, the results obtained so far cannot be directly translated to explain the surface activity of mAbs.

Currently, there are no simulation studies of complex multidomain proteins at the water/vapor interface. Here, we present all-atom MD simulations of mAb COE3, in both its native and slightly unfolded forms, at the water/vapor interface. We employ different force field /water model combinations and use these simulations to understand the relative importance of various energetic contributions to surface activity. Specifically, we (a) analyze the structural features associated with mAb adsorption, (b) identify the properties of adsorption–prone regions, and (c) quantify the structural changes incorporated into the mAb structure following interfacial adsorption.

## Materials and Methods

### mAb Models

We simulate an IgG1-based mAb, COE3, which has a Fc domain identical in sequence to the human IgG B12 (PDB id: 1HZH),^38^ whereas the Fab domain has a 73% similarity to the 1HZH Fab. The initial structure of the mAb was obtained from a recent paper by Singh and co-workers.^39^ The charges of various titrable amino acid (*aa*) residues in the sequence of the mAb were calculated at pH = 6 using propKa3.1.^40,41^

At pH 6, many HIS and GLU *aa*’s are protonated. The positions of these *aa*’s are shown in Figure S1 of the Supporting Information. The p*K*_*a*_ values for different titrable *aa*’s obtained from propKa analysis correspond to a total charge of +36 units for the mAb at pH = 6. The N– and C–termini were associated with a charge of +1e and −1e, respectively, which is the expected charge of the termini at pH = 6.

### Local Unfolding of mAb Using Thermal Stress

In addition to using the native structure of the mAb as the starting structure for our simulations, to address the impact of local structural changes on antibody adsorption at the water/vapor interface, we subjected the protein to thermal stress and generated a mildly unfolded variant of the native structure. The thermal stress led to local conformational changes at the protein surface that would not be observed under normal simulation conditions at 300 K. The local unfolding of the mAb structure was achieved by subjecting the mAb solvated in bulk water to a short MD simulation at a temperature of 450 K. We monitored the structural changes in the protein by calculating the RMSD of the mAb as a function of time (see Figure S2 of the Supporting Information). We selected a protein structure corresponding to a RMSD (with respect to the initial reference structure) value of 2.5 nm as the alternative starting structure. In addition to local structural changes, the enhanced interdomain flexibility of the mAb at higher temperatures results in more significant fluctuations of the interdomain distances, contributing to the total RMSD.

By isolating the contribution to RMSD from the fluctuations in the interdomain distance for the selected structure, we obtain a RMSD of 0.77 and 0.67 nm for the two Fab domains and 0.55 nm for the Fc domain relative to the native structure. The values of the RMSD of the different mAb domains quantify the degree of structural deformation of the mAb with respect to the starting structure due to thermal stress.

**Figure 1:**
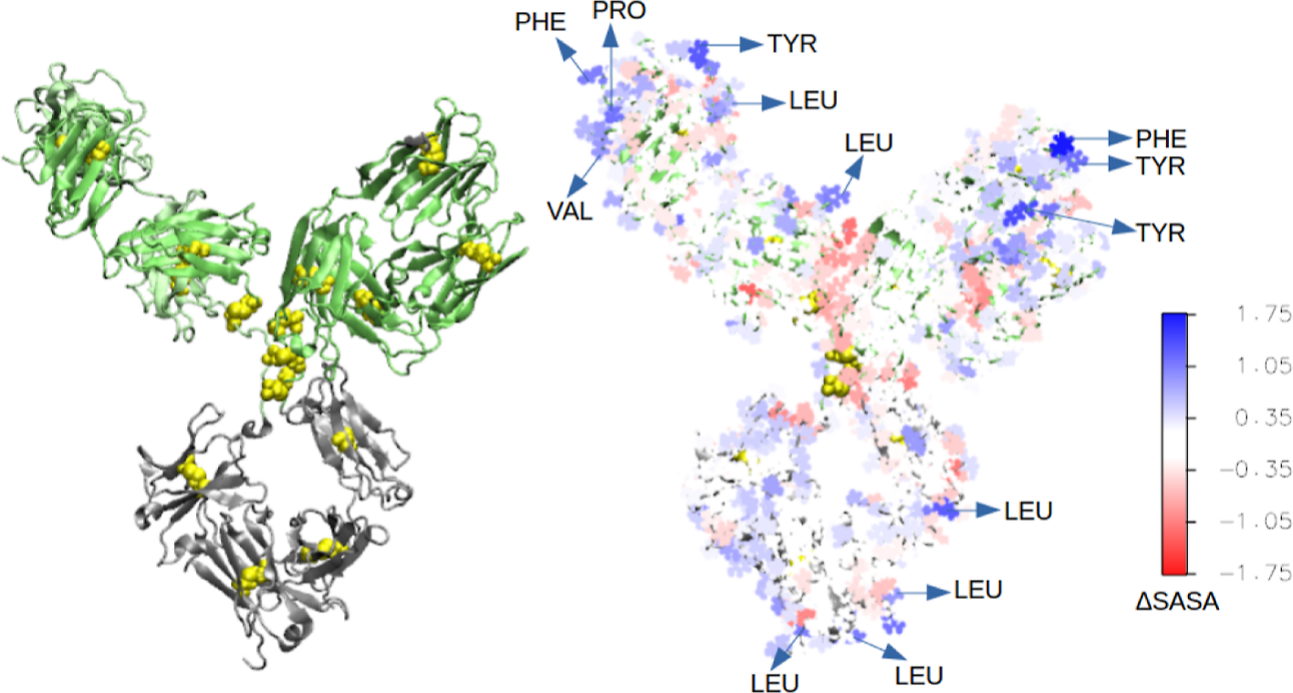
(left) Structure of the unfolded mAb (Fab domains shown in green and the Fc domain in silver). Cystines involved in disulfide bonds are shown in yellow. On the right is a surface color plot with the amino acid residues of the mAb based on the Δ*S*ASA value of the residue. Δ*S*ASA is calculated as the difference between the SASA of the residue in the unfolded and the folded mAbs. Δ*S*ASA_*i*_ = SASA_*i*_^unfolded^ – SASA_*i*_^native^. Residues (*i*) with the largest positive values of Δ*S*ASA_i_ are indicated.

We measured the structural differences between the natively folded and mildly unfolded mAbs at the single *aa* level by calculating the change in the solvent accessible surface area (SASA), Δ*SASA*_*i*_ = *SASA*_*i*_^unfolded^ – *SASA*_*i*_^native^, for each *aa* residue *i* of the mAb. Figure 1 shows the structure of the locally disrupted mAb. The residues are colored according to their respective Δ*S*ASA values, showing significant changes in the local structure of the unfolded mAb with respect to the native structure. Many hydrophobic *aa*’s (namely, LEU, TYR, PRO, PHE, and VAL) are among the residues with the largest positive values of Δ*S*ASA. This indicates that thermal stress leads to the exposure of hydrophobic residues buried in the interior of the natively folded mAb.

In addition to the natively folded structure and the specific unfolded mAb structure generated in bulk with thermal stress, we used simulated annealing (SA)^42,43^ to induce the disruption of the structure for the mAb adsorbed at the water surface. The natively folded mAb was used as the starting structure for these simulations. We subjected the mAb to heating and cooling cycles, between 300 and 400 K, followed by cooling to 300 K. This cycle was performed within a time interval of 1 ns. After the heating–cooling cycle, 4 ns of MD simulation was performed at 300 K (see Figure S3 of the Supporting Information), and the heating–cooling + MD cycle was extended for 200 ns. Throughout the 200 ns long simulation, the solvent temperature was fixed to 300 K. This approach resulted in slightly denatured protein conformations, with each heating–cooling cycle producing a slightly different unfolded conformation. All of these simulations were used for the analysis of protein adsorption as a way to identify adsorption “hot spots” on the mAb surface. Similar (but milder) temperature excursions have been used in experiments to study mAb particle formation in solution.^33^ We have added in the Supporting Information a short description explaining the advantages of the method used in this study to generate slightly unfolded proteins (see Figures S2 and S3 and associated text) over other possible methods.

We have performed simulations employing different protein force field (*ff*)–water model (*wm*) combinations and subjecting the protein to different simulation conditions. The systems simulated in this work have been named using the scheme: *ff*_*wm*_^condition^. As discussed above, the simulation conditions vary from using a native (no superindex in the system name) or slightly unfolded (indicated by superindex *unf*) starting structure of the protein generated using thermal stress and via simulated annealing (indicated by superindex *SA*) for generating mildly unfolded protein conformations during the simulation. The TIPs3P, TIP4P-2005, SPC, and SPC/E *wm*s are indicated by the subindices 3*P*, 4*P*, *SPC*, and *SPCE*, respectively. The section “Simulation Protocol” in the Supporting Information contains additional details on the simulation approach employed in this work, information on the simulation conditions for different systems (Table S1 of the Supporting Information), and a short discussion comparing the different *ff*s and *wm*s (Table S2 of Supporting Information) used in this work.

## Results

### Computer Simulation of Surface Activity of the Natively Folded mAb

**Figure 2:**
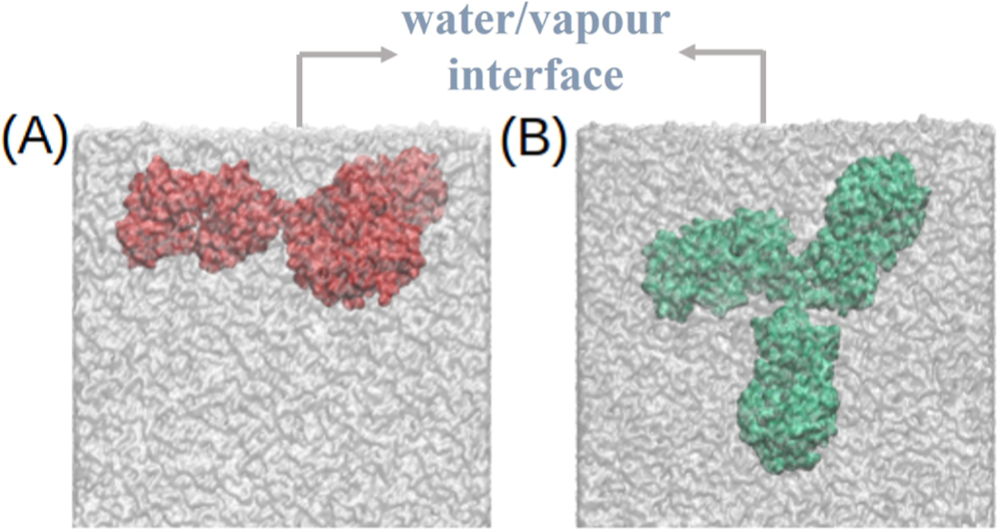
Final conformations from the (A) Charmm27_3*P*_ and (B) Charmm36_3*P*_ systems showing complete immersion of the protein in water. Only one of the two water–vapor interfaces is shown for each system.

To understand the microscopic mechanism of mAb adsorption, we performed all-atom simulations with widely used protein force fields. This analysis is particularly important since there are no previous simulations of mAb–water surface interactions. We started our analysis of the surface activity of the mAb using the Charmm27^44^*ff* (Charmm27_3*P*_; see Table S1 of the Supporting Information). This force field has been widely used and tested to investigate biomolecules and is known to reproduce their experimental behavior accurately.^45^ Our simulations showed intermittent adsorption of hydrophobic (Phe, Tyr, and Ile) and polar (Thr, Ser, and Asn) residues in the Fab domain at the water surface. The adsorption occurred only in the early stages of the simulation. After ∼50 ns, the native mAb submerged in the aqueous phase and stayed fully solvated during the rest of the 200 ns long simulation. This result indicates that the mAb modeled with Charmm27 *ff*(44) is not surface active. We performed additional simulations with Charmm36m *ff* (Charmm36m_3*P*_), which provides an improvement over Charmm27 *ff* in the sampling of the backbone and side chain dihedrals of proteins. However, the Charmm36m mAb did not adsorb at the interface either. Figure 2 shows the position of the mAb relative to the interface after 100 ns for both the Charmm27_3*P*_ and Charmm36m_3*P*_ systems. In both cases, the mAb is fully immersed in the water phase.

The adsorption free energy of a protein depends on the surface tension (γ_*w*_) of the water/vapor interface. To analyze the impact of γ_*w*_, we performed simulations using the TIP4P-2005 water model,^46^ which predicts γ_*w*_ in better agreement with experiments^47^ as compared to 3-point water models like TIPs3P. The value of γ_*w*_ of TIP4P-2005 water at 300 K for a 1 nm short-range cutoff that we use in our simulations is 62 ± 1.2 mN/m, much higher than the TIPs3P *wm* (50.3 ± 0.6 mN/m) at the same temperature (see Table S3 of the Supporting Information). Interestingly, our simulations with TIP4P-2005 *wm* and Charmm36m parameters for the protein (Charmm36m_4*P*_) predict mAb adsorption at the water surface. To quantify the degree of surface activity of the mAb, we computed the area of the mAb surface protruding out of the water into the vapor phase (*A*_*ads*_). We used the double-cubic lattice method introduced by Eisenhaber et al.^48^ The calculations were performed with a probe radius of 0.2 nm. The probe radius was selected to match the the result from previous calculations of the water surface roughness. The latter is defined as the ratio of the surface area of the pure water surface to the cross-sectional area of the simulation box. A probe radius of 0.2 nm provided the correct value for the surface roughness of water at the water/vapor interface.^49^ Time-averaged surface areas (*A*_*ads*_) are shown in Figure 4 (numerical values are listed in Table S4 of the Supporting Information). The time series of *A*_*ads*_ for all the *ff*/*wm* combinations are shown in Figure S5 of the Supporting Information. For the Charmm simulations performed with TIPs3P *wm* (Charmm27_3*P*_ and Charmm36m_3*P*_), hydrophobic regions of the Fab domain adsorbed transiently at the water surface for around 50 ns, but ultimately, for long simulation times ∼200 ns, *A*_*ads*_ reached a value of 0, indicating the full wetting of the protein. For the Charmm simulations performed with TIP4P-2005 *A*_*ads*_ = 7.0 nm^2^ (averaged over the last 150 ns of the trajectory), which indicates surface activity. The adsorption of mAb with the TIP4P-2005 model would be consistent with the higher surface tension of this model (Table S3 of the Supporting Information).

To further understand the adsorption observed with the TIP4P-2005 water model, we performed additional MD simulations of the Fab and Fc fragments and the complete mAb COE3 in bulk TIP4P-2005 and TIPs3P water. The *R*_*g*_ values of the Fc fragment and the mAb are significantly larger in TIP4P-2005 water than in TIPs3P water (see Figure S6 of the Supporting Information). The larger *R*_*g*_ originates from flexible regions in the protein, which acquire more extended conformations in the TIP4P-2005 water. The Fab domain, which lacks highly flexible regions, did not show significant changes in the *R*_*g*_ for the two *wm*s. While the higher surface tension of TIP4P-2005 *wm* will have a role to play in the observed adsorption of the mAb, the change in protein structure will also contribute. The less compact protein structure would result in a larger exposure of adsorption-prone regions on the mAb surface and, consequently, stronger adsorption of the mAb at the water/vapor interface.

We examine further the impact of the water surface tension and water–protein interaction by performing additional simulations with the Gromos96 54a7 *ff*(50) and the SPC water model, used in the initial parametrization of this *ff*. Gromos96 was developed by targeting solvation free energies, whereas the Charmm *ff*s use a combination of quantum mechanically and experimentally derived molecular geometries, vibrational data, pure solvent properties, and also free energies of solvation as the target data for parametrization.^51^ We find that Gromos *ff* predicts significant interfacial adsorption (see Figure S5 of the Supporting Information), as indicated by the large adsorption area (*A*_*ads*_) of ∼26 nm^2^. Interestingly, the SPC water model used in the Gromos *ff* model predicts a low surface tension, γ_*w*_ = 48.6 ± 0.5 mN/m (see Table S3 of the Supporting Information), even lower than the TIPs3P model employed with the Charmm *ff*, for which we did not observe adsorption. This result indicates that the water surface tension might not be the primary driver for the protein adsorption discussed above. Instead, protein–water interaction might play a role in determining the adsorption. This notion is supported by the radial distribution functions (RDFs) presented in Figure S7. The RDFs show a significantly weaker hydration shell for SPC than the TIPs3P or TIP4P-2005 models (see also the cumulative distributions as a function of radial distance in Figure S7). Recently, we measured and computed the second virial coefficient of the interaction between mAb COE3 fragments.^52^ The Gromos *ff* overpredicts attraction between Fc–Fc fragments and predicts attraction between Fab–Fab fragments, contradicting the repulsive interactions obtained in the experiments. However, Charmm *ff* predicts results consistent with the experiments.^52^ We infer from the results obtained in the present work and previous studies that the Gromos parameters impart a higher hydrophobicity to the protein surface in comparison to the Charmm *ff*s. This explains the stronger protein adsorption we observe at the water surface.

Based on the results discussed above, we conclude that there is no simple relationship between protein adsorption and water surface tension. While surface tension may play a role, the strength of water–protein interactions appears to have a significant impact on adsorption. An increase of 10–20 mN/m in the surface tension across water models does not lead to a monotonous increase in the protein adsorbed area. We note that protein distortion and, therefore, local changes in water–protein interaction lead to different adsorption behavior. Hence, we conclude that the water–protein interaction contributes, along with the liquid surface tension to the observed adsorption behavior. Modification of the strength of the water–protein interaction, e.g., through structural changes or chemical modification of the mAb surface, could provide a route to control the adsorption behavior of proteins. We analyze this idea in the following.

### Impact of Local Unfolding on mAb Adsorption

The results discussed above for the state-of-the-art Charmm *ff*s disagree with experimental studies of mAb COE3 using neutron reflectivity and experiments performed for other mAbs using various experimental techniques.^21,24−29,53^ These experiments provided clear evidence for the adsorption of mAbs at the water–vapor interface. However, other factors could explain the lack of protein adsorption observed with Charmm *ff*. Proteins in solution^54−56^ and interfaces^28,57^ might undergo local reversible or irreversible structural changes, such as disruption of the protein secondary structure. Irreversible changes might be more likely at liquid–vapor interfaces as the protein environment undergoes an abrupt change in hydration over a length scale of a few nanometers. Indeed, there is evidence that local changes in protein hydration can trigger local denaturation.^58^

To understand the impact of local structural changes on mAb adsorption, we performed simulations of a modified (slightly unfolded) mAb (referred to as *unf*) featuring locally unfolded regions. We quantified the degree of unfolding using the RMSD and Δ*S*ASA as described (see the Materials and Methods Section for details on how this configuration was generated and the analyses performed). We also quantified the degree of unfolding using the spatial aggregation propensity (SAP) index,^59−61^ which quantifies the solvent-exposed hydrophobicity of a protein. The SAP for an atom *j* belonging to a protein can be calculated using the equation^59,60^
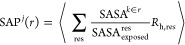
1
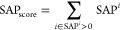
2where the sum runs over all the residues (res) with at least one side chain atom within a distance *r* (taken here as 0.5 nm), from the atom *j*. The brackets indicate a time average. SASA^*k*∈*r*^ is the solvent-accessible area of side-chain atom *k*, belonging to amino acid residue res, that lies within a distance *r* from atom *j*, and SASA_exposed_^res^ is the combined SASA of all the side-chain atoms in residue res. SASA_exposed_^res^ is, as a convention, computed by using the Ala–res–Ala trimer in bulk water. The values were taken from our earlier work,^61^ where we calculated the time-averaged SASA_exposed_^res^ from 50 ns long simulations of Ala–res–Ala trimers for all 20 *aa*’s (see Table S5 of the Supporting Information). *R*_h,res_ is the residue hydrophobicity following the Black and Mold (BM) scale.^62^ The BM scale is shifted such that *R*_h,GLY_ = 0 (see Table S5 of the Supporting Information). Thus, for a given atom, *j*, the SAP^*j*^ is a sum of the total hydrophobicity in the region surrounding the atom, weighted by a SASA-dependent factor that quantifies the exposure of that region to the solvent, hence providing a measure of the solvent-exposed hydrophobicity around the atom. A +*ve* (−*ve*) value of SAP implies a net hydrophobic (hydrophilic) environment on the protein surface in the region located around a given atom. The residue SAP is the average of all of the constituent atoms’ SAPs. The SAP score of a protein is then defined as the sum of SAP values over all residues with SAP > 0 (see eq 2).

**Figure 3:**
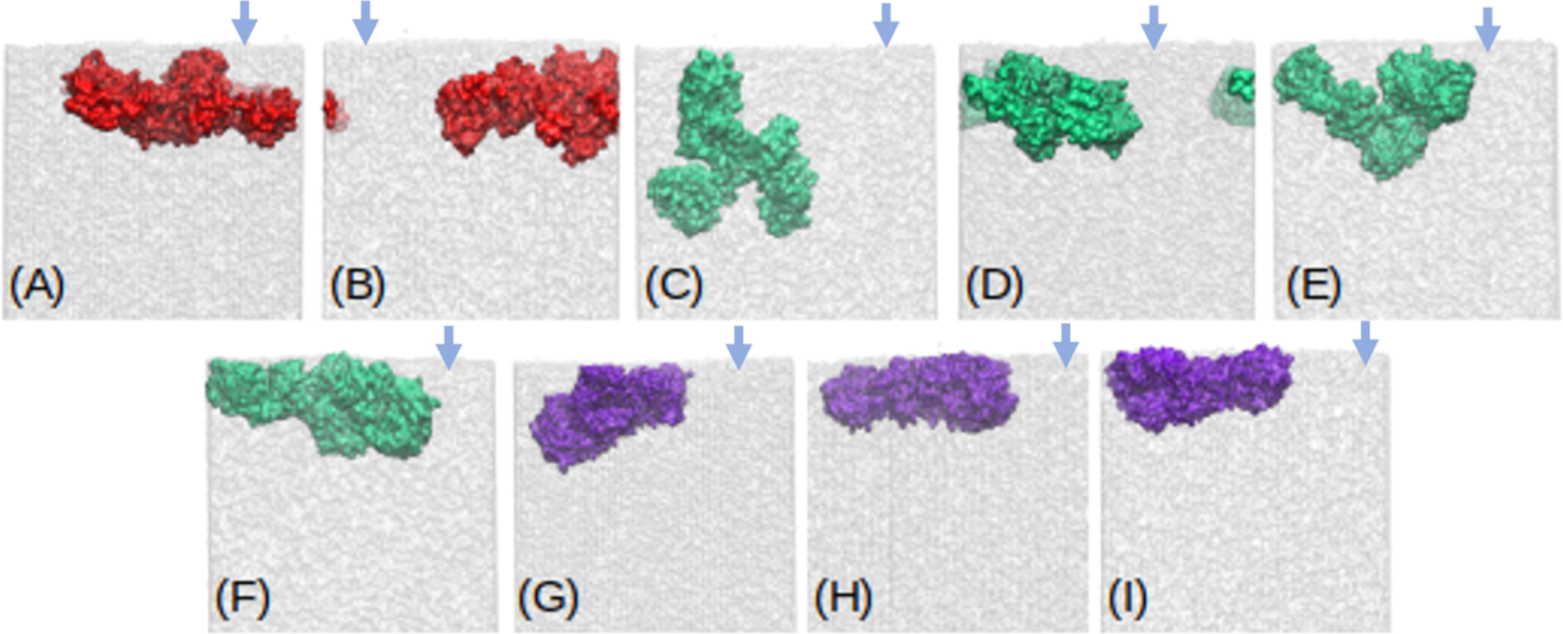
mAb at the water/vapor interface for (A) Charmm27 with unfolded mAb, (B) Charmm27 system with SA, (C) Charmm36m with unfolded mAb, (D) Charmm36m with SA, (E) Charmm36m with TIP4P-2005 water, (F) Charmm36m with TIP4P-2005 water model and SA, (G) Gromos, (H) Gromos with SA, and (I) Gromos with the SPC/E water model and SA. Arrow indicates the location of the water/vapor interface. Only one of the two water/vapor interfaces has been shown for each system.

**Figure 4:**
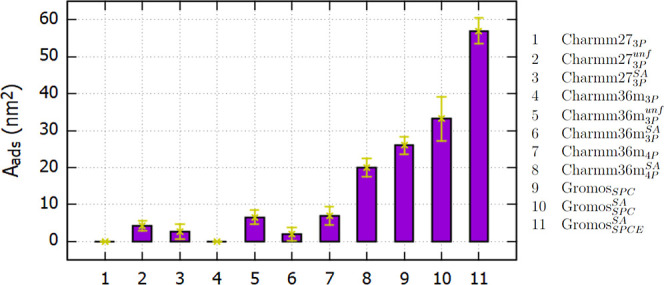
Surface area of mAb exposed to the vapor phase (*A*_*ads*_), averaged over the last 150 ns of the 200 ns long trajectories. The subindices 3P, 4P, SPC, and SPC/E indicate the water model (TIPs3P, TIP4P-2005, SPC, and SPC/E) employed in each simulation. The superindices *unf* and *SA* refer to the unfolded and simulated annealing generated COE3 mAb configurations discussed in the text.

**Figure 5:**
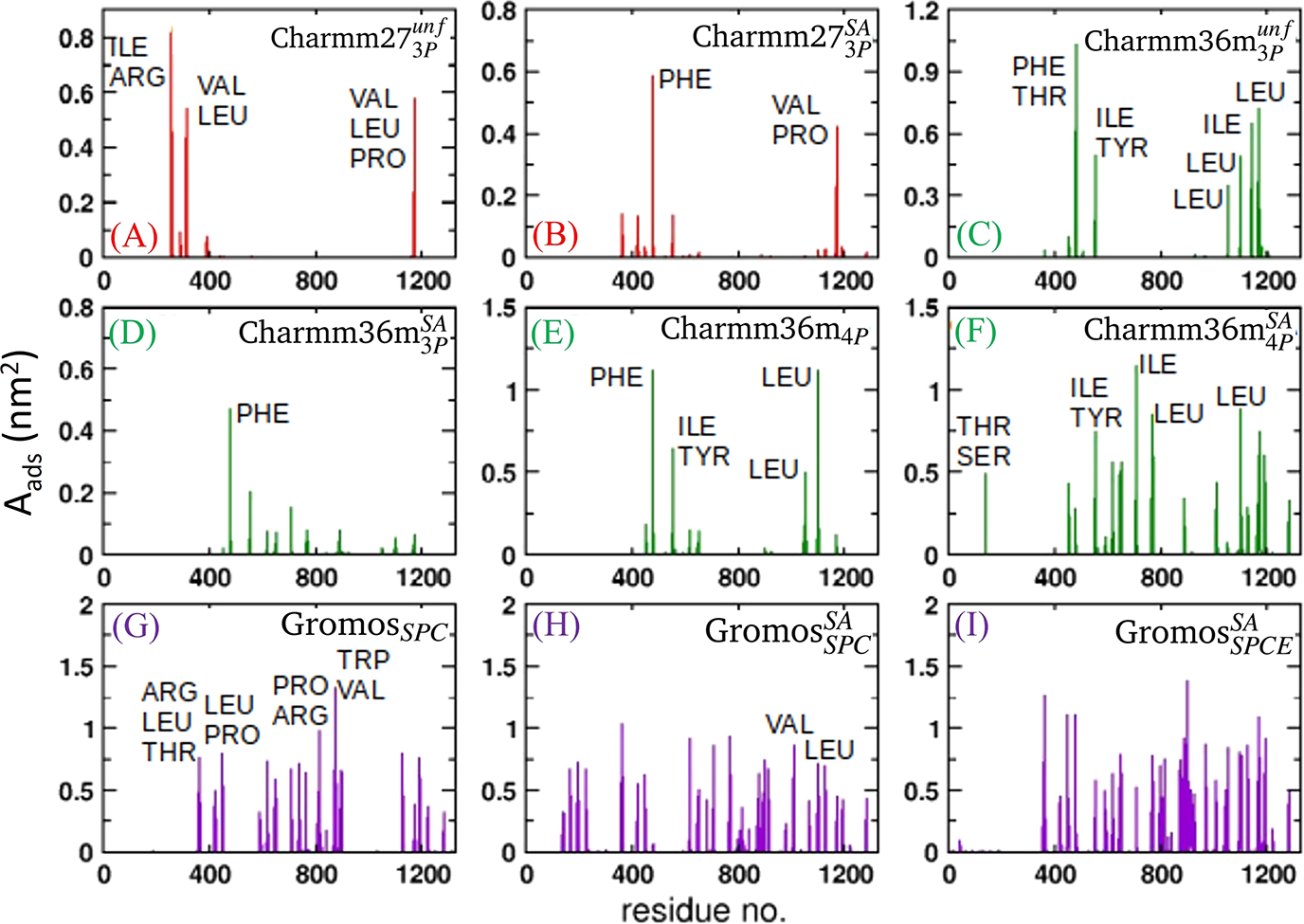
Residue area protruding into the vapor phase for the different systems simulated in this work. Areas have been calculated using the last 150 ns of the 200 ns long trajectories. Unfolded and simulated annealing generated conformations are indicated with the *unf* and *SA* superindex, respectively. Results without superindex refers to native protein structures.

The SAP score of the native mAb was found to be 31.6, while that of the starting configuration of the unfolded mAb (for the unfolded mAb simulations) was 39.2, indicating a higher solvent-accessibility/exposure of the hydrophobic regions of the mAb. Hence, the local unfolding results in a mAb that is globally more hydrophobic. The unfolded mAb simulated with both Charmm27 (Charmm27_3*P*_^*unf*^) and Charmm36m (Charmm36m_3*P*_^*unf*^) *ffs* show surface activity with *A*_*ads*_ values of 4.2 and 6.6 nm^2^, respectively (see Figure 3A,C and cases 2 and 5 in Figure 4). In addition to the total *A*_*ads*_ of the mAb, we calculated the *A*_*ads*_ for each *aa* belonging to the mAb. Figure 5 shows the residue-wise *A*_*ads*_ for each system. All *aa*’s with *A*_*ads*_ > 0 adsorb at the interface for a significant part of the trajectory. We see from Figure 5A,C that most of the *aa*’s with a high value of *A*_*ads*_ are hydrophobic. Hence, hydrophobic *aa*’s drive surface activity. To establish the correlation between interfacial adsorption and distortion of the mAb structure due to thermal stress, we calculated Δ*S*ASA for the protein regions adsorbing at the interface in the *unf* simulations (see Figure 1).

The Δ*S*ASA (see Materials and Methods Section for definition) for regions adsorbing at the water surface is 1.8 nm^2^ for the Charmm27_3*P*_^*unf*^ system and 7.1 nm^2^ for the Charmm36m_3*P*_^unf^ system, indicating a correlation between protein regions distorted by the thermal stress with the protein regions adsorbing at the water surface.

The simulations of slightly unfolded mAb discussed above provide a connection between the mAb surface activity and local denaturation. However, simulations starting from a single unfolded structure might not provide a full representation of the intermittent structural distortions that would appear due to thermal fluctuations in a therapeutic formulation and, therefore, it is not easy to infer the general impact of the mAb structural perturbations on adsorption and surface activity. To address this issue, we subjected the natively folded mAb to SA cycles (see Materials and Methods and Figure S4). By perturbing the protein structure periodically, we generated a wider range of mildly unfolded configurations on the fly. The process would lead to intermittent mAb structures with slightly more solvent-exposed hydrophobic *aa*’s as compared with the natively folded mAb during the course of the simulation. By design, the change in the exposed area compared to that of the native structure is minimal (see Figure S8 of the Supporting Information). The SA simulations using Charmm27 (Charmm27_3*P*_^*SA*^) and Charmm36m (Charmm36m_3*P*_^*SA*^) *ff*s predict mAb adsorption, revealing the effect of mild structural deformation of the mAb on interfacial adsorption. Compared with the Charmm27_3*P*_^*unf*^ and Charmm36m_3*P*_^*unf*^ systems, the SA configurations feature slightly weaker adsorption (c.f. surface areas in Figure 4). We infer from Figure 5 that the number of *aa*’s “pinning” the interface is smaller for the SA simulations (see Figure 5A–D). The impact of SA is more evident for the Charmm36m_4*P*_^*SA*^ system, which involves the TIP4P-2005 water model. This protein features a significant adsorbed area (*A*_*ads*_ = 20 nm^2^) (see system 8 in Figure 4), much larger than the *A*_*ads*_ observed with the native mAb conformation (Charmm36m_4*P*_, *A*_*ads*_ = 7.0 nm^2^, system 7 in Figure 4). The surface active *aa’s* of the protein simulated with the SA technique are distributed throughout the entire mAb surface (see Figure 5F), in contrast with the more localized aa patches observed in the native structure (see Figure 5E).

We performed SA simulations using Gromos *ff* as well. The impact of SA can be appreciated by comparing the results of Gromos_*SPC*_ (Figure 3G) for the native protein and Gromos _*SPC*_^*SA*^ (Figure 3H). In the latter case, the thermal stress leads to the adsorption of the protein surface with residues 900–1100 (c.f Figure 5G,H), which is surface inactive in the native structure. Additional SA computations were performed using the Gromos *ff* combined with the SPC/E *wm*(63−65) instead of the original SPC water. SPC/E water model is known to reproduce the hydration structure around hydrophobic *aa’s* better than SPC and, consequently, the hydration free energies are more accurate.^35^ The mAb adsorption shows an additional enhancement for the Gromos _*SPCE*_^*SA*^ system (see Figure S5). The large number of residues involved in the adsorption for the Gromos simulations span both the Fab and Fc fragments and contribute to the flat orientation of the mAb at the water surface as compared to a tilted one when the native structure is considered (see Figure 3G–I). From the RDFs shown in Figure S7, we infer that the SPC and SPC/E water models have the weakest interaction with the protein surface. This result further supports our view that the surface activity is strongly dependent on the water–protein interaction.

Our computations show that the Charmm *ff*s do not predict adsorption for the native mAb COE3 structure, but local unfolding induced by thermal stress leads to interfacial adsorption. While the strength of the water–protein interaction is found to be a major factor determining adsorption, it is worth exploring the energy of adsorption and the effect of local unfolding with respect to the water surface tension. The gain in free energy associated with the removal of water surface resulting from the protein pinning at the interface is substantial, As is in the range 24–80 (see data for Charmm27_3*P*_^*SA*,*unf*^ and Charmm36m_3*P*_^*SA*,*unf*^ systems in Figure 4). We find similar free energy changes for simulations performed with *unf* and TIPs3P and the native structure with TIP4P-2005 water. The SA structure in TIP4P-2005 features a large increase in the adsorbed area (see data for Charmm36m_4*P*_^*SA*^ in Figure 4), corresponding to . This enhanced adsorption is similar to the one obtained with the Gromos *ff* using the native structure (*A*_*ads*_ = 26.0 nm^2^ or ), where the neteffect of the force-field hydration free energy, water surface tension,^35^ results in a large protein area in the vapor phase. The adsorption is further enhanced when SPC is replaced by SPC/E, due to the better description of the water structure around hydrophobic molecules.^35^ The local unfolding results in an enhancement of the protein adsorption, which is maximized when using SA (with Gromos), resulting in a significant free energy change . The numbers above provide insight into the surface energy changes associated with the different protein structures investigated in this work.

**Figure 6:**
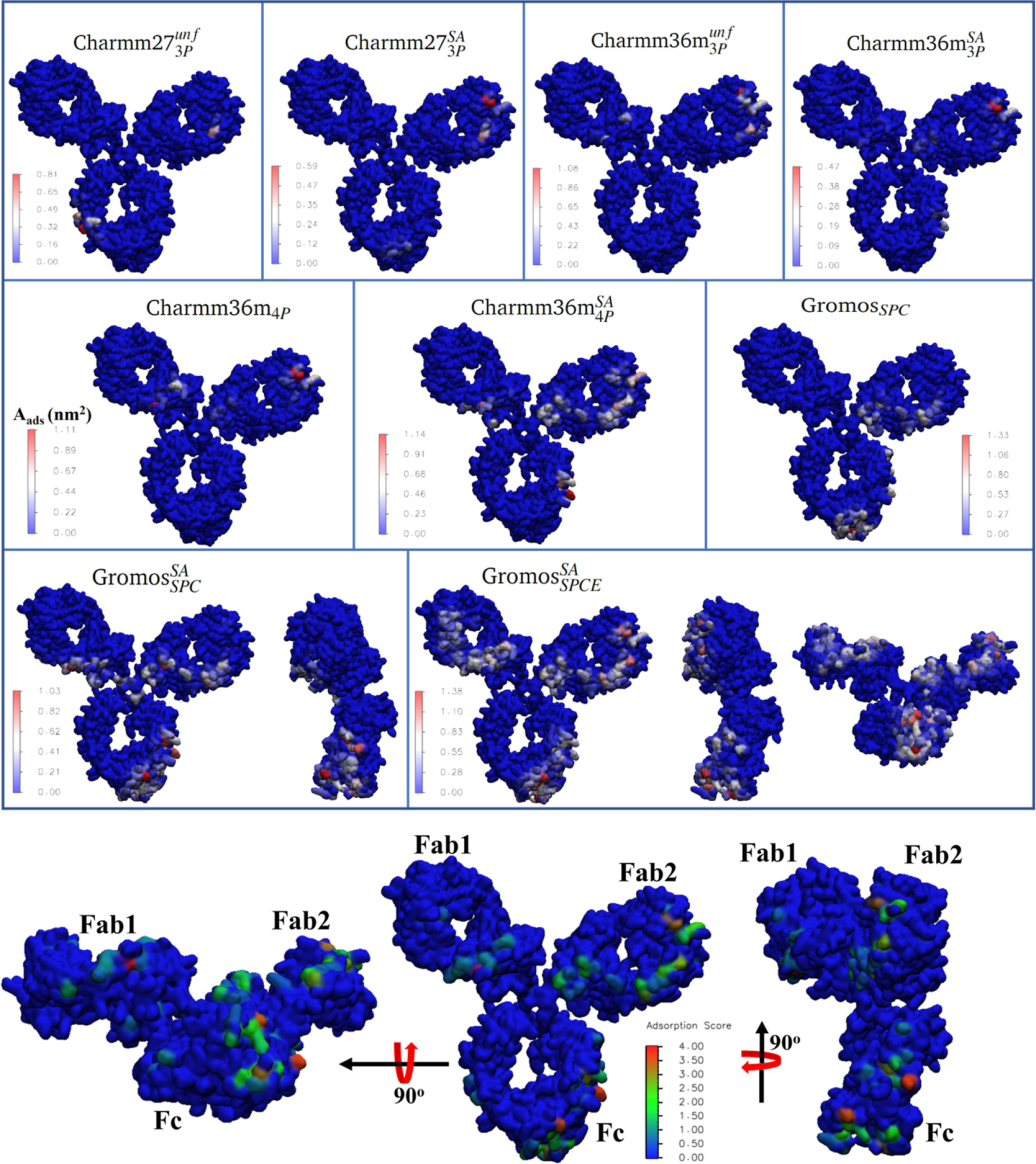
(Top 9 panels): surface of mAbs with *aa*′*s* colored by the value of their *A*_*ads*_. (Bottom three panels) surface of the mAb is colored according to the adsorption score (*S*) (eq 3) of different amino acids. The amino acids colored in red feature the highest adsorption score, followed by yellow and green. Blue color in this part represents amino acids with *A*_*ads*_ < 0.5 nm^2^.

Figure 3 illustrates the various adsorption modes of mAb at the water surface. Different parameters and simulation conditions result in different protein orientations relative to the interface plane. Weak pinning of the protein at the interface, corresponding to small *A*_*ads*_, is achieved with the plane of the mAb acquiring a slightly tilted to perpendicular orientation (see Figure 3A,B,D,E) with respect to the plane of the interface, with pinning proceeding via the Fab fragment (see also Figure 6). Strong adsorption invariably involves a flat mAb structure with both Fab and Fc fragments participating in the adsorption. This flat conformation agrees with the one reported in neutron reflectivity experiments of mAb COE3 performed in ref (25) and with the conclusion in that work that the short axial length of the mAb is perpendicular to the interface.

### Impact of Surface Adsorption on mAb Conformation

In this section, we compare the conformation of the mAbs at the interface with that in bulk solution. In our previous study,^61^ we investigated COE3 in bulk using the Charmm27 *ff* and the TIPs3P water model in 150 mM salt. The radius of gyration varies in the range of 4.2–5.75 nm with an average value of 5.0 ± 0.32 nm, which is close to the reported value of ∼5 nm obtained by SAXS measurements^66^ for the IgG1 subclass. The surface active mAb simulated in the Charmm27_3*P*_^*unf*^ and Charmm27_3*P*_^*SA*^ systems features slightly different sizes, *R*_*g*_ of 4.83 ± 0.06 nm and 4.67 ± 0.05 nm, respectively. Similarly, the mAb simulated in the Charmm36m_3*P*_^*unf*^ and Charmm36m_3*P*_^*SA*^ systems (where we observed adsorption) has an *R*_*g*_ of 4.52 ± 0.08 and 4.68 ± 0.06 nm, respectively. To establish a proper comparison, we calculated the *R*_*g*_ of the Charmm36m_3*P*_ system, which does not feature adsorption. The *R*_*g*_ of mAb in this system is 4.8 ± 0.3 nm, close to Charmm27 *ff*. Thus, the *R*_*g*_ of surface active mAbs simulated with Charmm36m *ff* and the corresponding standard deviations are much lower than those in bulk. This result indicates that adsorption reduces the average size and flexibility of the mAbs compared to that in solution. We can investigate this notion further by considering the Charmm36m simulations with the TIP4P-2005 water. The simulations predict surface activity and *R*_*g*_ = 5.15 ± 0.22 nm (Charmm36m_4*P*_) and 4.77 ± 0.16 nm (Charmm36m_4*P*_^*SA*^). The *R*_*g*_ obtained from simulations performed in bulk solution using the same parameters is 5.25 ± 0.44 nm. Again, the protein size and magnitude of structural fluctuations obtained with this model are lower than those obtained with the same *ff* with the mAb in bulk solution. These observations support the notion that mAb adsorption results in a stiffer protein than the protein in bulk solution.

### Identification of Surface Active Regions of the mAb

The surface active regions of the mAb for different systems are shown in Figure 6, with *aa* residues colored according to their respective *A*_*ads*_. The surface active regions are located on both the Fab and Fc domains. The simulations with the Charmm *ff* feature a sparse distribution of residues with non-zeroA_ads_, with the surface active residues located in similar regions of the mAb for conformations generated through heat stress (*unf*) or those generated using *SA*. However, there are clear differences in the active site distribution of the mAb simulated with the TIP4P-2005 model and SA, with a significant enhancement of surface active *aa*’s in the Fab region approaching the hinge. The mAb simulated with the Gromos *ff* features an otherwise significant amount of surface active regions, spread across the Fab and Fc fragments. This residue distribution favors the flat conformation shown in Figure 3H,I, which is consistent with the conformation inferred from the analysis of neutron reflectivity experiments.

To identify the surface active regions on the protein surface that have a strong tendency to adsorb, we use a residue-level adsorption score (), which is defined as the average *A*_*ads*_ of an amino acid over the 9 simulations where we observed adsorption (see top 9 panels of Figure 6). A particular value of *A*_*ads*_ contributes to the average only if it is larger than 0.5 nm^2^. Thus,
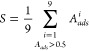
3The value of 0.5 nm^2^ has been chosen as the cutoff as it is close to the solvent-exposed area of a Gly residue (which has the smallest side chain among all *aa*’s) in an Ala–Gly–Ala tripeptide (the Ala–X–Ala tripeptide is a typical sequence motif used to calculate the exposed surface area of any *aa* species, X, for the calculation of parameters like SAP that are a measure of solvent-exposed hydrophobicity; see Table S5 of the Supporting Information). Figure 6 (bottom panel) shows the mAb surface with each residue colored according to our adsorption score,  (eq 3). The figure depicts the regions that drive the adsorption of the mAb. Such information can be used to modify the mAb in specific regions (by performing mutations, for instance) in order to control the interfacial adsorption behavior of mAbs. Modification of such regions would, in effect, change the strength of water–protein interactions, leading to a change in adsorption behavior.

We ranked different *aa* species according to their contribution to mAb adsorption based on all 9 simulations where we observed surface activity. LEU has the largest contribution, followed by VAL, PRO, ILE, PHE, TYR, and TRP. In Figure S9, we show the percentage contribution of these *aa*′*s* among all the hydrophobic residues (identified by using the BM scale) that correspond to a value of *A*_*ads*_ > 0.5 and 0.1 nm^2^. The lower cutoff of 0.1 nm^2^ has been introduced to obtain information on the nature of *aa*’s showing mild adsorption, which would be missed by the 0.5 nm^2^ cutoff. We also calculated the percentage of different *aa* species among all of the residues (hydrophobic or hydrophilic) adsorbing at the interface (see Figure S10). The percentage occurrence of LEU and VAL in the COE3 sequence is similar (∼7%). However, the amount of LEU featuring a large adsorbed area, and therefore surface activity, is much higher (cf. LEU and VAL in Figure S10). The larger fraction of LEU at the water–air surface is not a trivial result following the protein structure. Indeed, we find that the cumulative SASA for all LEU, VAL, and PRO residues in the native mAb structure are 30.3, 20.3, and 45.6 nm^2^, respectively. Thus, while the SASA of LEU residues is 1.5 times that of VAL, the number of LEU adsorbing with *A*_*ads*_ > 0.5 nm^2^ is ∼2.5 times that for VAL (see Figure S10 of the Supporting Information). In addition, PRO, which has a larger cumulative SASA than VAL and LEU, contributes much less (10%, as compared to 32 and 13% for LEU and VAL, respectively) to the group of strongly adsorbing *aa*’s, highlighting the higher hydrophobicity of LEU and VAL as compared to PRO (see Table S5 of the Supporting Information).

We find that as we increase the cutoff area for determining surface activity from *A*_*ads*_ = 0.1 to 0.5 nm^2^, the relative amount of hydrophobic residues increases (see Figure S9 of the Supporting Information). To obtain these results, we used a combined set of the nine systems represented in Figure 6. This implies that while there are polar residues adsorbing at the interface, these residues feature low *A*_*ads*_ and, therefore, small exposure to the vapor phase. As expected, most of the contribution from the polar *aa*’s to adsorption occurs for the Gromos parameters. Hydrophilic *aa*’s that do protrude into the vapor phase (ARG, THR, and SER) have a minor contribution to the total adsorbed surface area. Among all of the *aa*′*s* that have an *A*_*ads*_ greater than 0.5 nm^2^, 73% are hydrophobic.

**Figure 7:**
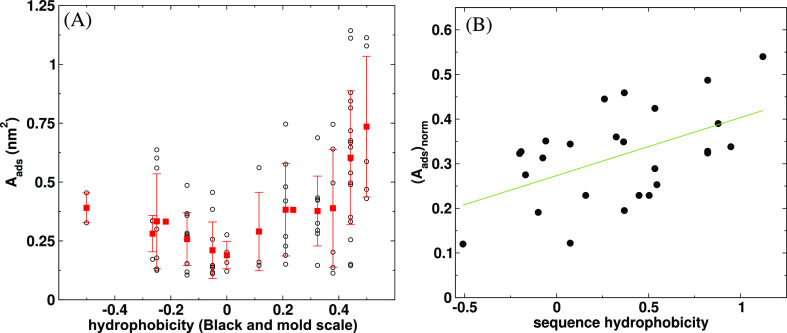
(A) Correlation between amino acid hydrophobicity^62^ and *A*_*ads*_ for all surface active amino acids with *A*_*ads*_ > 0.1 nm^2^. The black circles represent the *A*_*ads*_ for each residue and the red symbols with the error bar are the average and standard deviation for amino acids with the same hydrophobicity. (B) (*A*_*ads*_)_*norm*_ (see eq 4) as a function of sequence hydrophobicity for the sequences listed in Table 1.

We next analyze the correlation between the hydrophobicity and the *A*_*ads*_ for different *aa*’s adsorbing at the interface. In Figure 7A, we plot the *A*_*ads*_ for each *aa* (with *A*_*ads*_ > 0.1 nm^2^ from all simulations employing the Charmm *ff*s) as a function of its hydrophobicity (taken from the BM scale^62^). The adsorbed area, *A*_*ads*_, increases with the hydrophobicity of nonpolar *aa*’s. Similar behavior is observed for the *aa*’s showing interfacial adsorption in the Gromos simulations (see Figure S13).

In Table 1, we compile all the short *aa* sequences (2–8 *aa*’s long) found to adsorb at the interface in the Charmm simulations. We determined the correlations between the surface activity of specific amino acid sequences and their hydrophobicity (see Table 1). We introduce a normalized adsorption area 
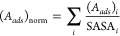
4whereas the sequence hydrophobicity is the sum of the hydrophobicities of individual *aa*’s constituting the sequence. Figure 7B shows the correlation between (*A*_*ads*_)_norm_ and the sequence hydrophobicity, with a higher cumulative hydrophobicity leading to stronger surface activity. This result suggests that while hydrophobic *aa*’s drive adsorption, the adsorption process is cooperative, and the presence of other hydrophobic *aa*’s in the neighborhood leads to stronger adsorption. We find that the cooperative effects lead to the adsorption of many hydrophilic *aa*’s like SER and ARG at the interface due to their proximity to hydrophobic *aa*’s (see Table 1).

**Table 1:** Amino Acid Sequences Adsorb at the Water Surface^a^

	system	adsorbing sequences
		*A*_*ads*_ (amino acid)> 0.1 nm^2^
1	Charmm27_3*P*_^*unf*^	**ILE SER ARG**
		VAL LEU HIS GLN, **VAL PRO**
2	ChaCharmm27_3*P*_^*SA*^	GLY VAL PRO
3	Charmm36m_3*P*_^*unf*^	**PHE THR PHE**
		ILE TYR, **SER SER LEU**
4	Charmm36m_4*P*_	**GLN VAL**, GLY PHE THR, **ILE TYR**
		THR GLN, **ASN ALA LEU GLN SER**
		LEU SER SER PRO
5	Charmm36m_4*P*_^*SA*^	**THR SER**, GLN VAL, **GLY PHE THR**
		ILE TYR, **ALA LEU THR SER**
		PRO SER SER SER LEU GLY THR GLN
		**LEU MET ILE**
		GLN ASP TRP LEU ASN
		**THR VAL ALA ALA**, LEU SER SER PRO
		**VAL GLY**
		LEU GLN SER GLY VAL PRO SER ARG
		**SER LEU GLN PRO**
		

a All the results were obtained with the Charmm force field. Figure 7B shows the correlation between the total hydrophobicity of the short sequences listed in the table and the normalized adsorbed area of the sequence per residue. To enhance readability, consecutive different sequences are represented by normal or boldface letters. Sequences that adsorb at the interface in simulations employing the Gromos *ff* are shown in Table S6 of the Supporting Information.

**Figure 8:**
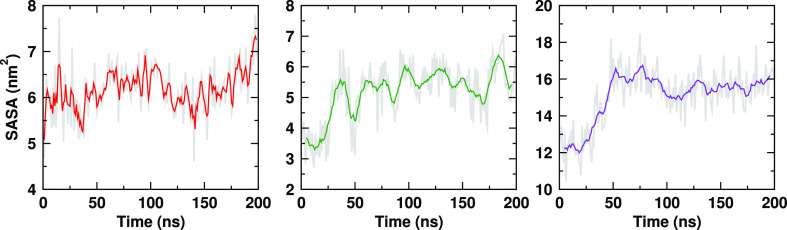
Time dependence of the total SASA of the surface active hydrophobic amino acids for (red) Charmm27_3*P*_^*unf*^, (green) Charmm36m_3*P*_^*unf*^, and (violet) Gromos_*SPC*_ systems. SASA values were calculated at 1 ns interval. Running average was taken over 10 consecutive data points.

We next examine the kinetics of the surface active regions of the mAb by computing the time dependence of the cumulative SASA of all the hydrophobic *aa* residues that adsorb at the interface. We find that this SASA features a tendency to increase with time, indicating that the hydrophobic aa’s undergo an enhancement of their exposed areas at the water surface (Figure 8). On the contrary, the cumulative SASA of all the hydrophobic *aa*’s (adsorbed at the interface or not) in the mAb decreases with time (see Figure S11). This result supports the notion that the mAb regions adsorbed at the interface behave in a manner distinct from that in the bulk. The regions evolve dynamically and possibly are prone to disruption, reflected in a concomitant enhancement of the exposed hydrophobicity of the regions that adsorb at the interface. These observations agree with previous experimental studies by Leiske et al.,^67^ which reported an increase in the surface hydrophobicity of mAbs adsorbed at the water–vapor interface. Thus, while small structural perturbations appear to be a prerequisite for interfacial adsorption, the regions adsorbed at the interface seem to undergo further structural changes. In an experimental setup, irreversible local structural deformations can be incorporated due to adsorption at the water surface. Such locally deformed mAbs, on detachment from the surface, might contribute to mAb aggregation in formulations.^68^

**Figure 9:**
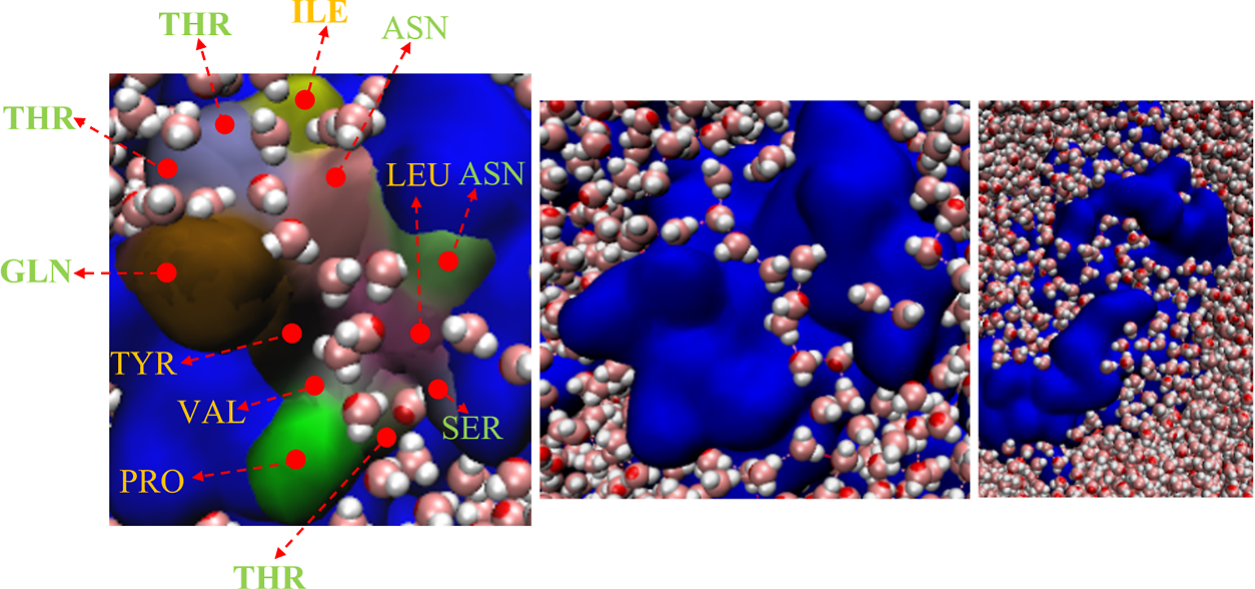
Transient water “wires” in the hydrophilic ridges on the protein surface in the vapor phase (middle and right panels). The amino acids forming one of the ridges in the middle panel are highlighted and labeled in the left panel. The hydrophobic amino acids are labeled in orange, while the hydrophilic ones are labeled in green. All snapshots were extracted from Charmm36m_4*p*_ trajectory.

We conclude this section by providing additional microscopic insight into the regions that determine the adsorption area, *A*_*ads*_. The mAb adsorbed at the water/vapor interface forms small aqueous islands on the water surface. We find intermittent water structures that cover transiently the protein surface. Figure 9 shows two snapshots from the MD trajectories obtained usingCharmm36m_4*P*_^*SA*^. The snapshots show the presence of water “wires” connected via hydrogen bonds and spanning narrow regions on the protein surface. The formation of these water structures is stabilized by the presence of polar *aa*’s at the surface, which is pulled to the water surface by the neighboring hydrophobic residues (see Figure 9-left panel). The intermittent water structures mostly interact with the polar regions.

The interaction of water adsorbed on surface active regions of the protein may resemble the interaction of water with mAb in the lyophilized state, where a small number of water molecules adsorb on the mAb surface. Feng et al.^69^ performed simulations of the lyophilized state of the mAb and found that the water molecules mostly adsorb on the polar regions of the mAb surface. We also find that the ridges formed between *aa* sequences host water chains and layers next to polar *aa*’s, such as ASN, THR, GLN, and SER. The information on the water surface structures shown in Figure 9 might be relevant to improving models used to fit neutron reflectivity profiles, particularly layer models that assign a single scattering length density value to a given layer.

## Discussion

We investigated the interaction of mAb COE3 with the water surface using all-atom MD simulations. Our simulations reveal a strong dependence on the adsorption behavior with the forcefield employed. State-of-the-art forcefields such as Charmm27 and Charmm36m do not predict the surface activity of native proteins. This behavior starkly contrasts with experiments, particularly reflectivity experiments,^25^ which showed clear evidence that the mAb investigated here is surface active at different protein concentrations and buffer conditions. Evidence for adsorption has also been found in other experimental studies.^21,24,26−29,53^ In this work, we examined other forcefields, such as Gromos, which is widely used by the biophysical community. This forcefield predicts strong mAb surface activity of the native mAb structure and a significant contribution to adsorption from hydrophobic and hydrophilic residues. This behavior aligns with previous studies of smaller proteins, such as lysozyme,^37^ which demonstrated that the adsorption process predicted by the Gromos forcefield is less sensitive to the *aa* composition (phobic vs philic) at the protein surface. These observations are correlated with an overestimation of hydration enthalpies of proteins^37^ and the overestimation of protein–protein attraction in mAb fragments, which highlight the enhanced “hydrophobicity” of this forcefield.^52^

We conclude that the inability of a forcefield to predict mAb adsorption is not necessarily connected to its inaccuracy. Instead, our work highlights the importance of local structural changes in the mAb when the protein is in contact with the water surface. Indeed, previous experimental studies indicate that protein adsorption might trigger a process that leads to the disruption of the protein structure. We tested the importance of the protein structural changes on adsorption by subjecting the proteins to thermal stress and SA. We find that the thermal stress induces small local structural changes, leading to an increase in the solvent-accessible surface area of hydrophobic *aa*’s, which leads to the adsorption of the slightly deformed mAb at the water surface. The enhancement of adsorption upon slight unfolding is observed for all the forcefields employed in this work. Even for the Gromos forcefield, which features the strongest protein hydrophobicity and adsorption for the natively folded mAb, the thermal stress induces adsorption enhancement. Thus, our results support local protein denaturation as a microscopic mechanism contributing to protein adsorption at the water surface. Wood et al.^70^ demonstrated that the adsorption of mAbs at the water–vapor interface at different concentrations follows a scaling characteristic of a diffusion-limited mechanism. Their experiments also show a bulk mAb concentration dependent rate of reduction of surface tension. Based on the Lumry–Eyring model^71−73^ of protein aggregation, distortion of the protein structure can occur due to the formation of long-lived protein–protein encounter complexes. Such encounter complexes are more likely to be present at higher mAb concentrations, which might lead to structural deformation and adsorption of a larger fraction of interface-bound mAbs compared to experiments performed at low bulk mAb concentrations. This might explain the higher rate of reduction of the surface tension at higher mAb concentrations. We believe that the slight structural distortions examined here might provide a route to facilitate protein adsorption.

We further demonstrate that the structure of initially unfolded mAb structures evolves, under thermal stress, by increasing the solvent-accessible surface area of hydrophobic residues, hence contributing to stronger adsorption.

We identified key regions of the mAb surface that drive adsorption. The surface active *aa* sequence are located in both the Fab and Fc domains. This favors the adsorption of mAb in a flat conformation, a result that agrees with the neutron reflectivity experimental analyses. However, the simulations also provide evidence for adsorption through other competing structures involving small regions of single fragments. This adsorption mechanism involves smaller areas on the mAb surface interacting with the water surface. Therefore, it is expected to be less energetically favorable than the flat conformation, where a significantly larger protein region participates in the adsorption process. We also found a correlation between the degree of hydrophobicity (as quantified by the BM hydrophobicity scale) and surface activity. Sequences containing the hydrophobic residue LEU feature stronger adsorption. In fact, we find that this residue is over-represented at the water surface, contributing over 40% of the *aa*’s adsorbed at the water surface, followed by VAL (∼15–20%) and PRO (∼10–15%). This is a notable result, since the amounts of LEU, VAL, and PRO in the COE3 sequences are very similar.

Our work provides conclusive evidence for the importance of local denaturation on mAb adsorption. The structural deformations mentioned here might be present under experimental conditions in solution and might well be a prerequisite for adsorption at the interface. In addition, by calculating the time dependence of SASA, we demonstrate that the regions adsorbed at the water/vapor interface tend to further distort. Hence, future theoretical/simulation work of mAb should include this effect, too, as the local native structure might not provide an optimal representation of the state of the mAb in contact with the water surface. A future extension of our work could consider different mAb structures to probe further structure/adsorption correlations in these important therapeutic proteins. Additionally, it will be interesting to increase the level of complexity of the simulation models by incorporating more mAbs to address the impact of cooperative adsorption effects and modification of the conformation of the proteins adsorbed at the water surface. This is important as the mAb concentration in the bulk also affects the orientation of the mAbs at the interface. To model more closely the experimental studies, specifically the solution composition of therapeutic formulations, buffer molecules could be included in future studies. These simulations might allow a more direct comparison with neutron reflectivity experiments, by simulating full mAb monolayers, and the potential cooperative effects emerging from protein–protein interactions, in the presence of buffer and excipients, such as those used in experiments, to understand their impact on the solvent accessibility of hydrophobic regions on the mAb surface (see, e.g., a paper from our group,^61^ for an analysis of mAb in bulk solutions containing histidine). The protocols described in this work can be used to study the effect of additional experimental variables, such as pH and ionic strength, on interfacial adsorption.

Another aspect that deserves attention is the mechanism regulating the adsorption process. To evaluate the reversibility of this process, one would need to consider the free energy associated with the detachment of the mAbs from the interface, including the differences between the interfacial and bulk free energies, as well as the activation free energy barriers. These aspects have been investigated before using spherical and anisotropic particles, with γ_w_A_ads_, being an important factor determining adsorption at the individual particle level within the thermodynamic theory of capillarity.^74,75^ An analysis of the reversibility of protein adsorption, incorporating the highly heterogeneous structure and surface interactions of proteins, would be a useful extension of the ideas presented in this work.

Finally, the simulation results presented here might provide a route to controlling mAb adsorption by modifying specific sequences in the mAb structure that drive adsorption. Ultimately, the modification of such sequences might offer better control of the mAb stability in biomedical applications.

## Supporting Information Available

Structure of mAb COE3; slightly unfolded mAb structures; description of the simulation protocol and a comparison of different *ff*s and *wm*s; *A*_*ads*_ as a function of time for the mAb for all the simulations; comparison of mAb structure for the TIPs3P and TIP4P-2005 *wm*s used in combination with the Charmm36m *ff*; γ_*w*_ of different *wm*s; *rdf* of water around the Fc fragment simulated in bulk for different *wm*s; cumulative SASA of hydrophobic amino acids as a function of time for the normal and SA simulations; time-averaged *A*_*ads*_ of the mAb for different simulations; hydrophobicity (BM scale) and bare SASA for all *aa*s; percentage composition of surface active *aa*s; variation of cumulative SASA of hydrophobic *aa*s as a function of time; variation of *R*_*g*_ as a function of time for simulations employing the Gromos *ff*; *A*_*ads*_ as a function of amino acid hydrophobicity; and list of surface active *aa* sequences from the different simulations employing the Gromos *ff* (PDF)
