# Supplementary material for: Mechanistic Insights into the Adsorption of Monoclonal Antibodies at the Water/Vapor Interface
Source: Mol Pharm. 2024 Jan 9;21(2):704–17. doi: 10.1021/acs.molpharmaceut.3c00821 (PMC10848294; doi:10.1021/acs.molpharmaceut.3c00821)
Supplement: Supplementary file 1 — mp3c00821_si_001.pdf [file mp3c00821_si_001.pdf]

Mechanistic insights into the adsorption of monoclonal antibodies at the water/vapor interface
Supplementary Information

Suman Saurabh,[†] Qinkun Zhang,[†] Zongyi Li,[‡] John M. Seddon,[†] Cavan Kalonia,[¶] Jian R. Lu,[‡] and Fernando Bresme[*,†]

†*Department of Chemistry, Molecular Sciences Research Hub Imperial College, W12 0BZ, London, United Kingdom*

‡*Biological Physics Group, School of Physics and Astronomy, Faculty of Science and Engineering, Oxford Road, The University of Manchester, Manchester M13 9PL, UK*

¶*Dosage Form Design and Development, BioPharmaceutical Development, BioPharmaceuticals R&D, AstraZeneca, Gaithersburg, Maryland 20878, United States*

E-mail: f.bresme@imperial.ac.uk

Phone: +44 207 594 5886

Protein

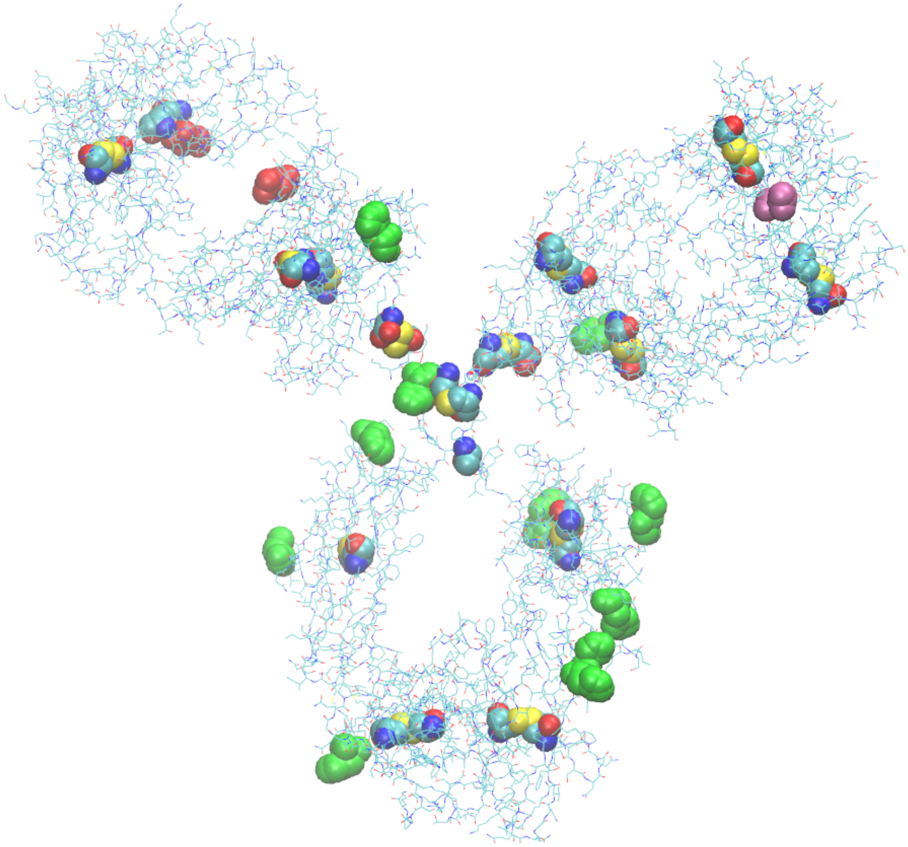

Figure S1: **The structure of mAb COE3. The amino acids shown in green, red and pink are the His, Asp and Glu residues that were protonated to mimic pH=6. The yellow beads correspond to the sulphur atoms linked by disulfide bonds, shown along with the corresponding Cys residues.**

Slightly unfolding the mAb structure

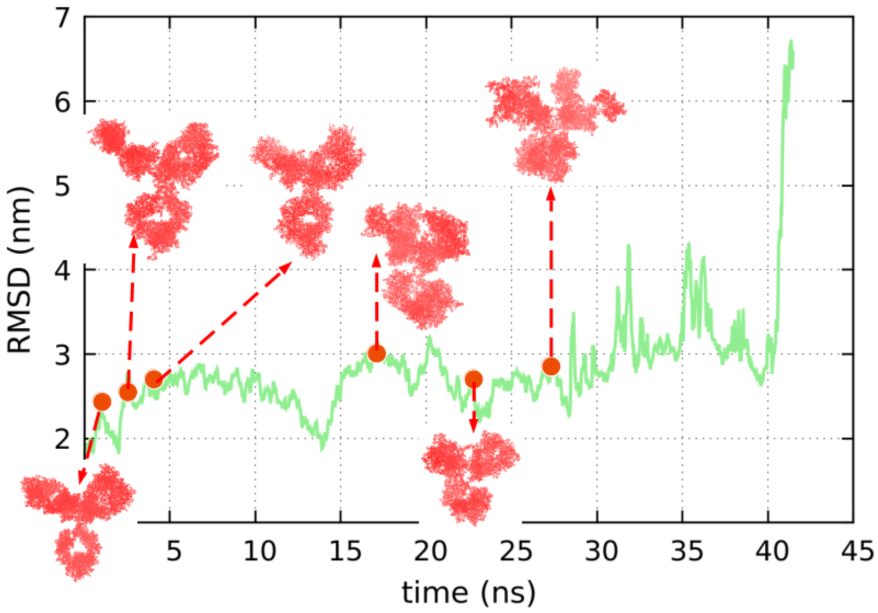

Figure S2: **The RMSD of COE3 as a function of time for the simulation performed at 450K using the Charmm27 ff and TIPs3P water model. The trajectory shows the formation of locally unfolded structures. The structure after 2 ns was chosen for the interfacial simulations presented in the main text.**

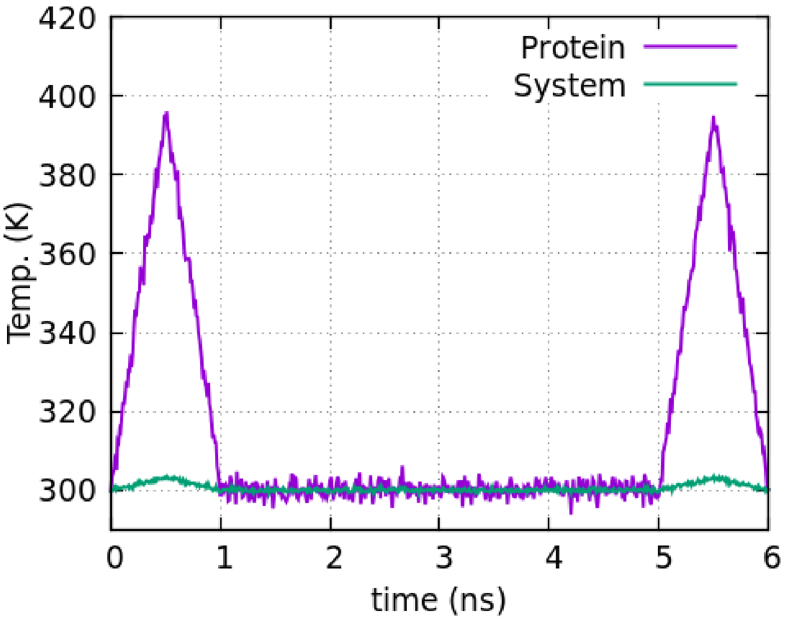

Figure S3: **Time dependence of the temperature for the protein and the whole system during a simulated annealing cycle.**

As discussed in the main text, we considered two approaches to generate slightly distorted mAb structures:

- Performing a short simulation in bulk water with the system heated up to a temperature of 450 K and choosing a slightly distorted structure (see figure S2) from the trajectory based on the RMSD with respect to the native mAB structure as the starting structure for further unbiased simulations.

- Generating mildly distorted structures "on the fly" by performing simulated annealing (SA) with the temperature of the mAb varying as shown in the temperature cycle in figure S3, with the cycle repeated multiple times upto the total simulation time of 200 ns.

In addition to these methods, there are other approaches that could possibly be used for inducing slight unfolding. Among them are methods like Replica Exchange MD (REMD),[1] Replica Exchange with Solute Tempering (REST),[2] and accelerated MD (aMD).[3]

In REMD,[1] simulations are performed at multiple temperatures (replicas) and conformations are exchanged between higher and lower temperature replicas based on a metropolis-like scheme. Due to small energy fluctuations in explicit solvent simulations (due to large system size), a large number of closely spaced temperature replicas are required for a significant exchange probability, leading to a very high computational cost.

REST[2] falls under the broader category of hamiltonian replica exchange techniques. These are modified forms of the original REMD where a modified system hamiltonian is used with rescaled solvent-solvent and solute-solvent interactions for the higher temperature replicas. The rescaling renders the exchange probability independent of solvent-solvent energy leading to a requirement of a smaller number of replicas. In the context of generating slightly unfolded mAbs, one can use REST by performing parallel simulations at two closely spaced temperature replicas (300 K and a higher temperature). Exchange of conformations of the system between the higher and lower temperature replicas would result in slightly

unfolded structures from the higher temperature replica then appear at 300K.

aMD[3] is another enhanced sampling technique where an additional term in the potential energy function leads to the "shallowing down" of local potential wells in the free energy landscape of the system, leading to an easier barrier jumping between free energy minima. This method can also be used to generate unfolded structures on the fly and has the advantage over REST that it can be used to only modify the free energy landscape of the solute, keeping the solvent untouched.

While the above-mentioned methods can be used in a simulation to generate mildly unfolded systems, our preferred method offered the following advantages:

- Techniques like REST may affect the structure of the interface in addition to the structure of the protein. Thus, after every exchange between the original (300K) and high temperature replicas, the system needs time for the equilibration of the interface, which is not required with the SA technique we used. The system temperature in SA differs only slightly from 300K (see figure S3).

- There are also control issues we wanted to avoid. In REMD, for instance, a temperature very close to 300K for the higher temperature replica may not generate unfolded structures, and a temperature very far from 300K would generate unphysical structures. For aMD on the other hand the parameters determining the modification in the free energy landscape need a very careful choice to control the unfolding level. For the SA technique, one can easily calibrate the degree of unfolding by changing the duration of the temperature shock and its magnitude.

- The method employed here does also mimic what would be employed in an experiment.

Simulation protocol

Table S1: **Water−vapour interface simulations (150 mM NaCl, no buffer) performed in this work. All the simulations have similar starting configurations for the mAb at the interface. 3P: TIPs3P, unf: unfolded, SA: Simulated annealing, 4P: TIP4P-2005**

	System	mAb	force field, water model	Temperature	Time	Adsorption?
1	Charmm27$_{3P}$	native	Charmm27, TIPs3P	300K	200ns	no
2	Charmm27$_{3P}^{unf}$	unfolded	Charmm27, TIPs3P	300K	200ns	yes
3	Charmm27$_{3P}^{SA}$	native	Charmm27, TIPs3P	SA	200ns	yes
4	Charmm36m$_{3P}$	native	Charmm36m, TIPs3P	300K	200ns	no
5	Charmm36m$_{3P}^{unf}$	unfolded	Charmm36m, TIPs3P	300K	200ns	yes
6	Charmm36m$_{3P}^{SA}$	native	Charmm36m, TIPs3P	SA	200ns	yes
7	Charmm36m$_{4P}$	native	Charmm36m, TIP4P-2005	300K	200ns	yes
8	Charmm36m$_{4P}^{SA}$	native	Charmm36m, TIP4P-2005	SA	200ns	yes, strong
9	Gromos$_{SPC}$	native	Gromos96 54a7, SPC	300K	200ns	yes, strong
10	Gromos$_{SPC}^{SA}$	native	Gromos96 54a7, SPC	SA	200ns	yes, strong
11	Gromos$_{SPCE}^{SA}$	native	Gromos96 54a7, SPC-E	SA	200ns	yes, Strong

We initially built water boxes with a size of 20 nm × 20 nm × 20 nm, using different water forcefields, TIPs3P, SPC, SPC/E and TIP4P-2005. The water boxes were minimized, using the steepest descent method, and subjected to a short 2 ns simulation and constant temperature and pressure using the v-rescale thermostat with a time constant of 0.5 ps and the Berendsen barostat with a time constant of 0.5 ps to allow the density to equilibrate.

After equilibration, the water boxes were placed at the centre of a box of size 20 nm × 20 nm × 40 nm elongated in the Z-direction, generating water slabs and 2 water/vapour interfaces. The mAb was placed at one of the interfaces partially immersed in water (see figure S4). The system was neutralized by replacing some water molecules with 36 Cl^{-} ions. In addition, 734 Na^{+} and an equal number of Cl^{-} ions were added to obtain a salt concentration of 150 mM.

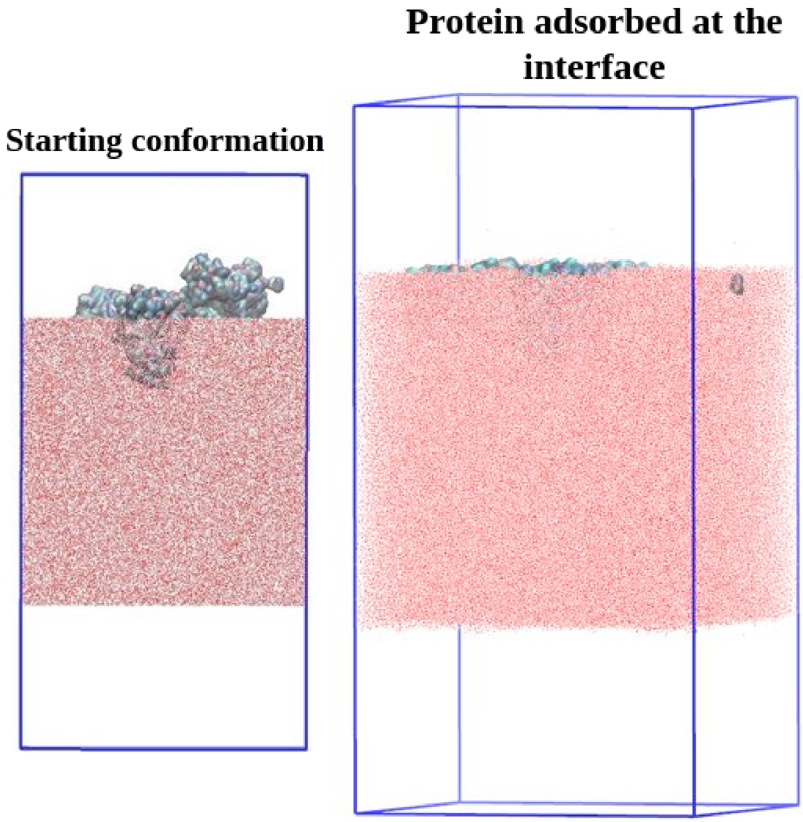

Figure S4: (Left) The starting configuration of the mAb at the water/vapour interface. (Right) A representative snapshot of the mAb, following equilibration, showing adsorption at the interface.

The systems were relaxed using the steepest descent method with all the protein atoms held fixed to their initial positions with harmonic restraints (force constant, 1000 kJ·mol^{-1}·nm^{-2}). Short 1 ns NVT simulations were performed at 300K with the protein atoms held to their initial positions. The canonical v-rescale thermostat[4] was used for temperature control with

a temperature coupling constant of 0.5 ps. Following this pre-equilibration process, we performed 200 ns long restraint-free production runs in the NVT ensemble.

All the simulations reported in this work were performed using the GROMACS(2021.3) software[5,6] package. Different force field (*ff*)-water model (*wm*) combinations were used to represent the mAb and the solvent. The simulation boxes are periodic in all three directions. The Particle Mesh Ewald method was used to compute the long-range electrostatic interactions, and the Lincs algorithm was employed to restrain intramolecular bonds involving hydrogen. All the simulations were performed using a time step of 2 fs. See table S1 for a list of all the simulations performed in this work.

Protein forcefields and water models used in this work

As listed in table S1, we have used 5 different protein *ff*-water model combinations in this work namely: 1. Charmm27+TIPs3P, 2. Charmm36m+TIPs3P, 3. Charmm36m+ TIP4P-2005, 4. Gromos96 54a7+spc and 5. Gromos96 54a7+ spce.

Charmm27 and Charmm36m *ff*s have been parameterized by using **experimental data** and data obtained from quantum calculations as the target data set, whereas the Gromos *ff*s generally use the experimental solvation free energies of amino acid analogs as the target data for parameterization. Charmm36m represents an improvement over Charmm27 *ff* and performs better in representing the balance between helical and extended conformations of proteins. Gromos *ff*s uses a united atom approach (hydrogen and carbon atoms merged into a single bead) for the non-polar bonds involving hydrogen atoms, whereas the Charmm *ff*s treat the non-polar hydrogens explicitly. The all-atom approach is expected to be more accurate, but in certain cases the united-atom approach leads to better results,[7] suggesting that *ff* parameters with higher complexity may not always lead to better results and the choice of forcefield depends on the problem at hand. This motivated our investigation of different forcefields, which rely on alternative strategies to model protein-water interactions.

The Charmm *ff*s have been parameterized with the TIPs3P water model whereas the

Gromos *ff*s with the spc water model

The water models used in this work differ in the way they represent the structure and interaction of the water molecules. The structural properties and interaction parameters of different water models are shown in table S2 The spc water model is a 3-point water

Table S2: **Parameters of water-models used in this work**

Model	H–O–H angle (^{o})	O–H (nm) bond length	q_H	q_O	q_{dummy}	σ_H (nm)	σ_O (nm)	ϵ_H (kJ/ mol)	ϵ_O (kJ/) mol)
TIPs3P	104.52	0.09572	0.417	-0.834	–	0.04	0.31506	0.1925	0.63639
spc	109.47	0.1	0.41	-0.82	–	0	0.31656	0	0.65019
spc/e	109.47	0.1	0.4238	-0.8476	–	0	0.31656	0	0.65019
TIP4P-2005	104.52	0.09572	0.5564	0	-1.1128	0	0.31589	0	0.77490

model, where the oxygen atom interacts with other atoms through both Lennard-Jones (LJ) and electrostatic interactions while the hydrogen atoms do not have Lennard-Jones interactions associated to them. The SPC/E model is a modified version of the SPC model with polarization corrections incorporated. The TIPs3P water model is also a 3-point water model, but differs from the SPC model in both structure (H–O–H angle and O–H) and interaction parameters. The model assigns LJ centers on the hydrogen atoms too. The TIP4P-2005 model is a 4-point water model, where the H atoms act as charge centers with no LJ interaction. The O atom acts as a LJ center, and a fourth dummy interaction site along the bisector of the H–O–H bond, acts as the negative charge center.

Following the observations with protein *ff*s, no single water model reproduces all the experimental properties of water. Generally, a protein *ff* is usually combined with the water model that is used for its parameterization. We used in this study combinations of Charmm36m+TIP4P-2005 and Gromos96 54a7+spc/e, to test the behaviour of the mAbs in water models. In particular, TIP4P-2005, very accurately reproduces the surface tension of water while the SPC/E model reproduces the hydration structure around molecules better than SPC. Further information can be found in the references cited in the main text.

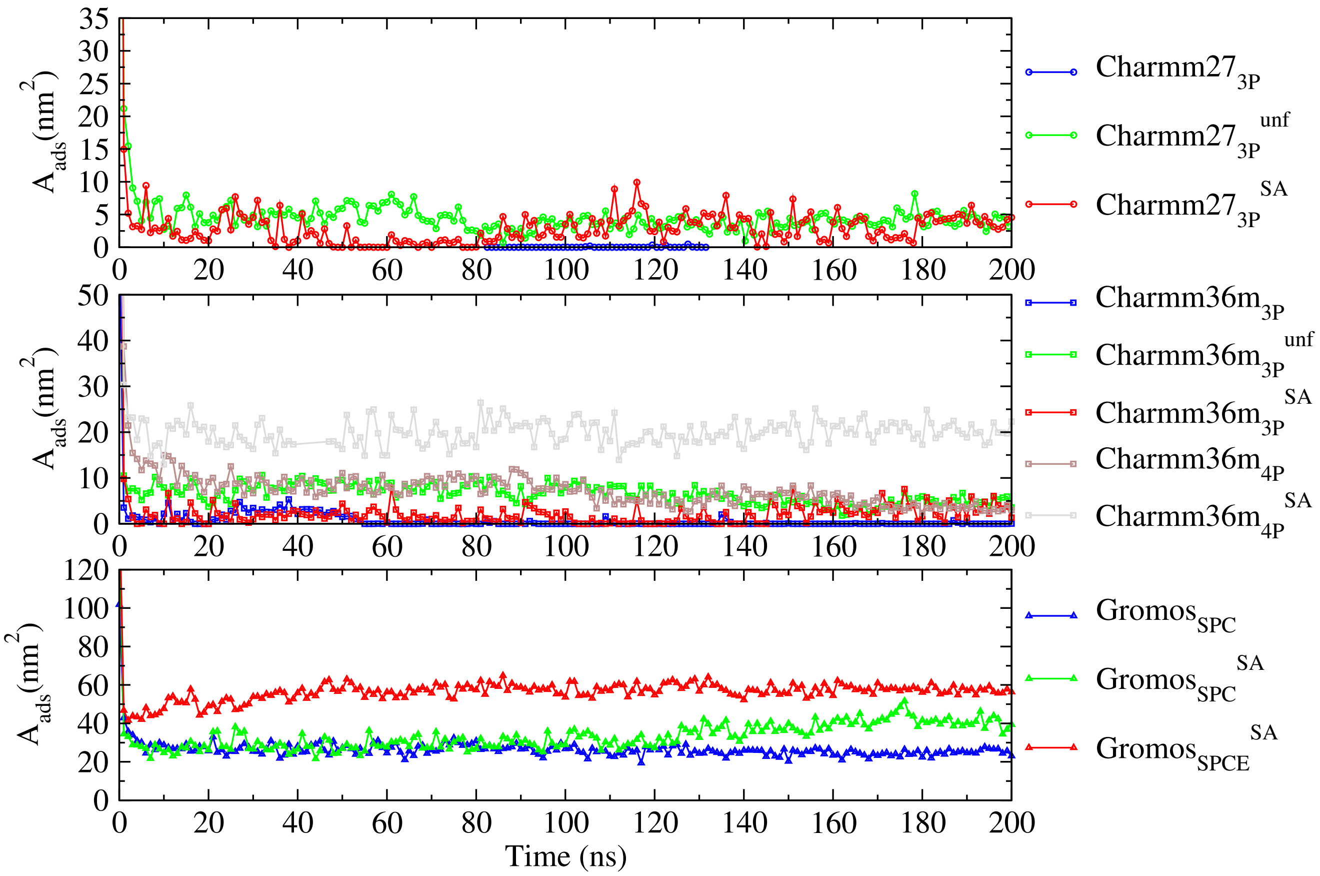

Figure S5: $\mathbf{A}_{ads}$ **vs. time for the different systems simulated in this work. Top, middle and bottom panels refer to Charmm27, Charmm36 and Gromos force-fields, respectively. See the caption of Table S1 for definitions.**

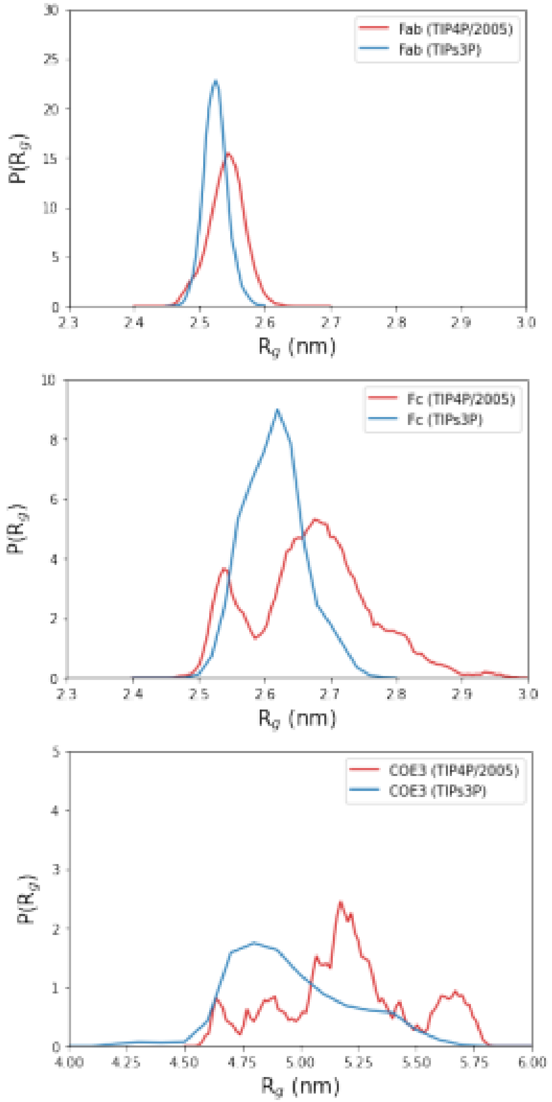

Figure S6: **Radius of gyration of the Fab and Fc fragments and the full antibody COE3 obtained using the TIPs3P and TIP4P-2005 water models in combination with the Charmm36m ff.**

Table S3: **Surface tensions of the water-models used in this work. The values have been calculated by averaging over equilibrated frames from 20 ns long MD simulations of an explicit water/vapour interface at 300K.**

	Water model	surface tension (mN/m)
1	TIPs3P	50.3 ± 0.6
2	SPC	48.6 ± 0.5
3	SPC-E	56.0 ± 0.2
4	TIP4P-2005	62.0 ± 1.2

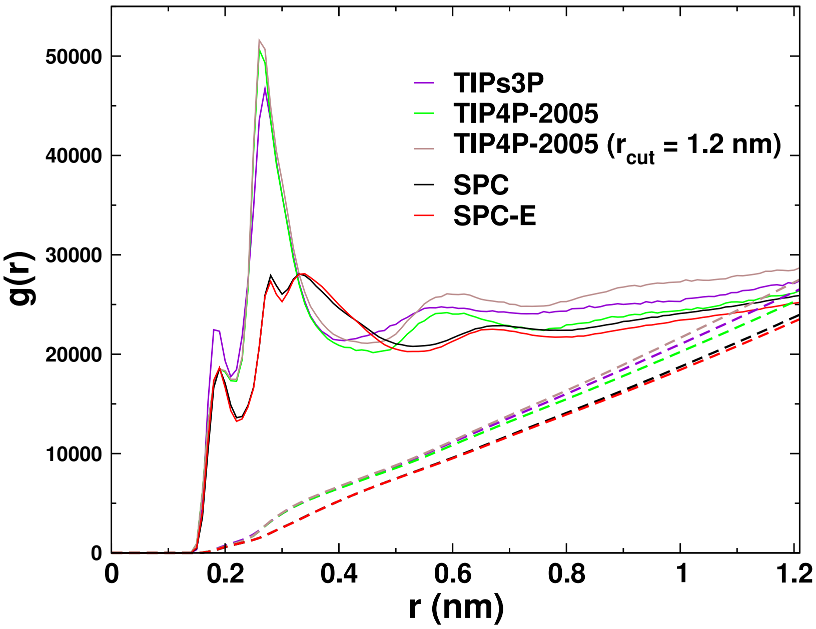

Figure S7: **The radial distribution function and the corresponding cumulative distribution (dashed lines) of water atoms around an Fc fragment for the TIPs3P, TIP4P-2005, SPC and SPC/E water models. The results have been obtained from 50-100 ns long simulations performed in bulk solution. The TIPs3P and TIP4P-2005 distributions have been obtained with the Charmm36m parameters for the protein, while the SPC and SPC/E results have been obtained with the Gromos96 54a7 parameters for the protein. All the simulations were performed with a r_{cut} = 1 nm unless otherwise stated. See reference[8] for the definition of the RDF represented here.**

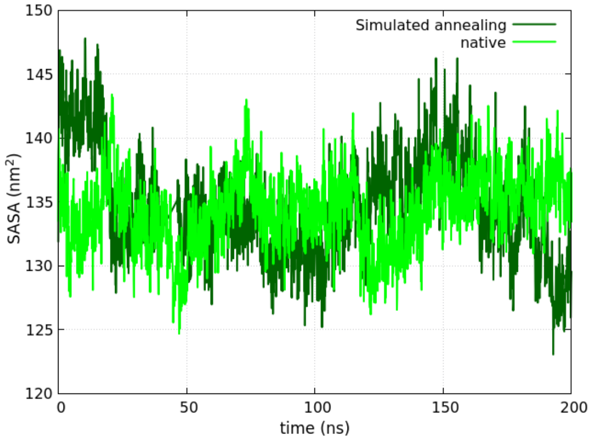

Figure S8: **Solvent accessible surface area of the hydrophobic amino acids of the mAb for Charmm36m$_{4P}$ and Charmm36m$_{4P}^{SA}$ systems. The SASA calculation excludes the reduction due to inter-domain contacts within the mAb. The SASA calculation was performed using *gmx sasa*. To exclude the effect of inter-domain contacts, an index file was generated with three separate atom groups including atom indices of the Fab1, Fab2 and Fc domains. In addition, three atom groups were created that contain the atom indices of the hydrophobic amino acids of Fab1, Fab2 and Fc domains. The SASA, without taking into account the inter-domain contacts, was then calculated by using the Fab1/Fab2/Fc groups as the input to the *-surface* flag and the corresponding group for hydrophobic amino acids as the input to the *-output* flag of *gmx sasa*.**

Table S4: **The surface area of mAb (A_{ads}) in the vapour phase for the systems simulated in this work. The sub-indices $3P$, $4P$, SPC, $SPCE$ indicate the water model (TIPs3P, TIP4P-2005, SPC, SPC/E) employed in each simulation. The superindices unf and SA refer to the unfolded and Simulated annealing COE3 configurations (discussed in the main text).**

	System	A_{ads} (nm^2)
1	Charmm27_{3P}	-
2	$\text{Charmm27}_{3P}^{unf}$	4.2 ± 1.4
3	$\text{Charmm27}_{3P}^{SA}$	2.7 ± 2.0
4	Charmm36m_{3P}	-
5	$\text{Charmm36m}_{3P}^{unf}$	6.6 ± 2.0
6	$\text{Charmm36m}_{3P}^{SA}$	2.0 ± 1.7
7	Charmm36m_{4P}	7.0 ± 2.5
8	$\text{Charmm36m}_{4P}^{SA}$	20.0 ± 2.5
9	Gromos_{SPC}	26.0 ± 2.4
10	Gromos_{SPC}^{SA}	33.2 ± 6.0
11	$\text{Gromos}_{SPCE}^{SA}$	57.0 ± 3.5

Table S5: **Amino acid hydrophobicity scale taken from the Black and Mould Scale, normalized such that Gly has a hydrophobicity of 0. A value > 0 implies a hydrophobic amino acid, while a value < 0 implies a hydrophilic amino acid. SAA$_{exposed}$ is the solvent accessible area (probe radius = 0.2 nm) of the sidechain for different central residues. The SAA of residue X was computed in pure water using the *Ala–X–Ala* trimer as described in the next section.**

**Amino acid**	**Hydrophobicity**	**SAA$_{exposed}$** (nm^2)
Ala	0.115	0.8819
Arg	-0.501	2.5819
Asn	-0.265	1.5714
Asp	-0.473	1.3939
Cys	0.179	1.2328
Gln	-0.250	1.9099
Glu	-0.458	1.7785
Gly	0.000	0.4446
His	-0.336	1.9039
Ile	0.442	1.8721
Leu	0.442	1.8722
Lys	-0.218	2.2582
Met	0.237	1.9684
Phe	0.499	2.2303
Pro	0.210	1.1826
Ser	-0.142	1.1436
Thr	-0.051	1.4026
Trp	0.377	2.6130
Tyr	0.379	2.3409
Val	0.324	1.5479

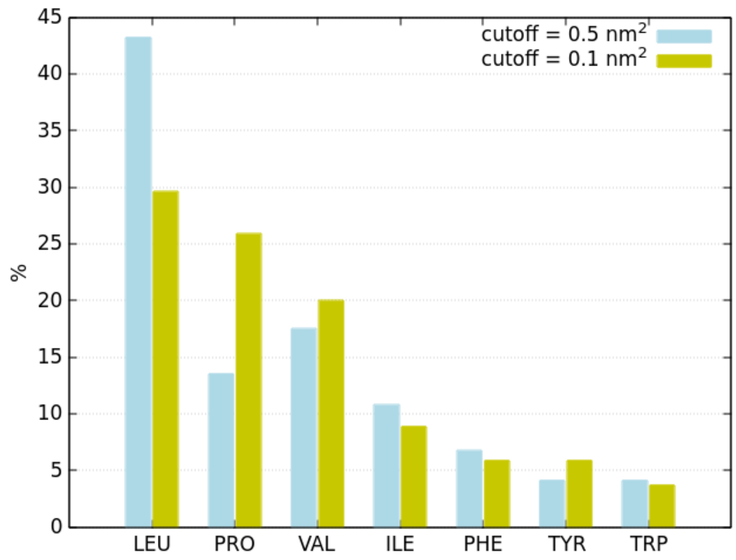

Figure S9: **The percentage of different hydrophobic amino acids with $\mathbf{A}_{ads} > 0.1$ or 0.5 nm^2 over all simulations. The percentages have been calculated over all hydrophobic amino acids that adsorb at the interface.**

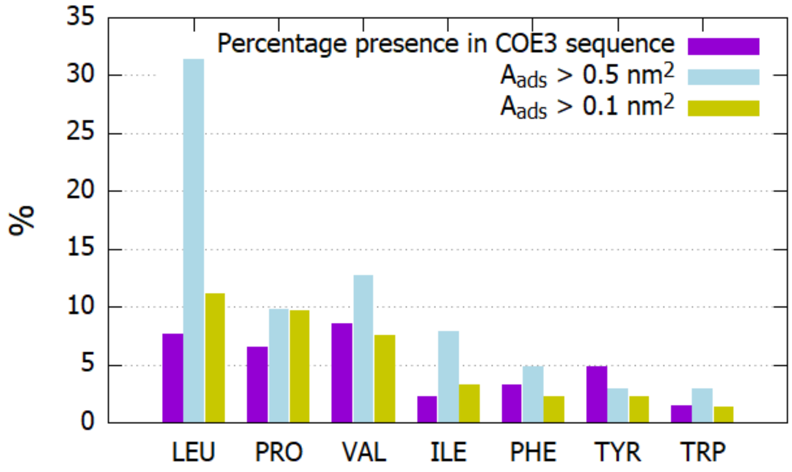

Figure S10: **Population (percentage) of amino acids with $\mathbf{A}_{ads} > 0.1$ or 0.5 nm^2, for all the simulations. The percentages have been calculated over all amino acids (hydrophobic or hydrophilic). The percentage population of the aminoacid species in the mAb sequence is also shown (purple bars).**

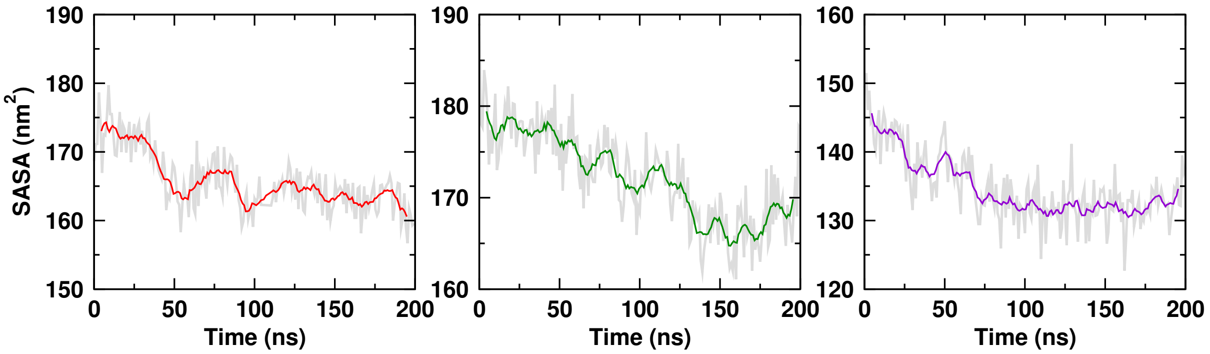

Figure S11: **Time evolution of the solvent accessible surface area (SASA) for all the hydrophobic amino acids in the mAb for the (red) (Charmm27$_{3P}^{unf}$), (green) (Charmm36m$_{3P}^{unf}$), and the (violet) (Gromos$_{SPC}$) systems. The SASA has been calculated by ignoring inter-domain contacts within the mAb and the figures show the time evolution purely due to local structural rearrangements (see caption for figure S8.**

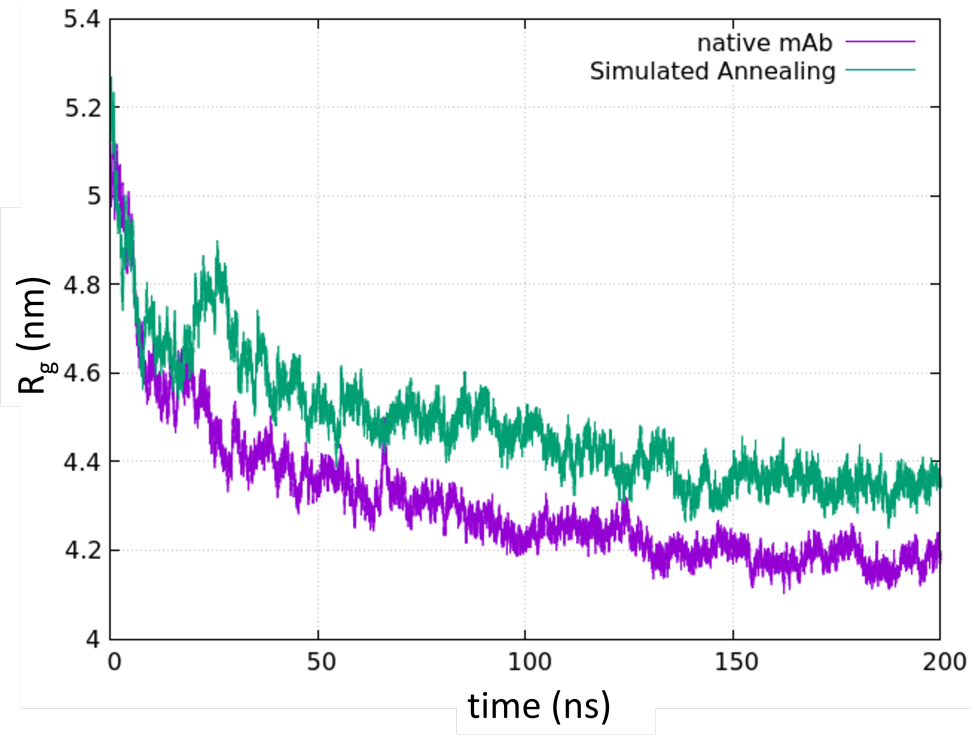

Figure S12: **The radius of gyration of COE3 as a function of time for the Gromos$_{SPC}$ (native) and Gromos$^{SA}_{SPC}$ systems.**

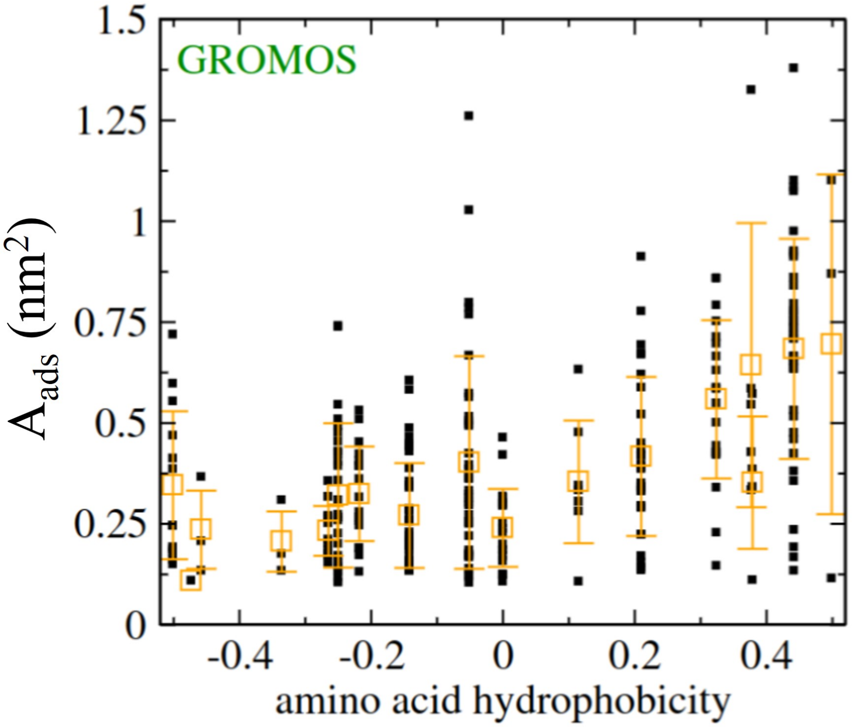

Figure S13: **Variation of A_{ads} with residue hydrophobicity for all amino acids with $A_{ads} > 0.1$ nm^2 taken from all the Gromos simulations performed in this work.**

References

(1) Bernardi, R. C.; Melo, M. C.; Schulten, K. Enhanced sampling techniques in molecular dynamics simulations of biological systems. *Biochimica et Biophysica Acta (BBA)-General Subjects* **2015**, *1850*, 872–877.

(2) Liu, P.; Kim, B.; Friesner, R. A.; Berne, B. J. Replica exchange with solute tempering: A method for sampling biological systems in explicit water. *Proceedings of the National Academy of Sciences* **2005**, *102*, 13749–13754.

(3) Hamelberg, D.; Mongan, J.; McCammon, J. A. Accelerated molecular dynamics: a promising and efficient simulation method for biomolecules. *The Journal of chemical physics* **2004**, *120*, 11919–11929.

(4) Bussi, G.; Donadio, D.; Parrinello, M. Canonical sampling through velocity rescaling. *Journal of Chemical Physics* **2007**, *126*, 014101.

(5) van der Spoel, D.; Lindahl, E.; Hess, B.; Groenhof, G.; Mark, A. E.; Berendsen, H. J. C. GROMACS: Fast, Flexible and Free. *Journal of Computational Chemistry* **2005**, *26*, 1701–1718.

(6) Abraham, M. J.; Murtola, T.; Schulz, R.; Páll, S.; Smith, J. C.; Hess, B.; Lindahl, E. GROMACS: High performance molecular simulations through multi-level parallelism from laptops to supercomputers. *SoftwareX* **2005**, 19–25.

(7) da Silva, G. C.; Silva, G. M.; Tavares, F. W.; Fleming, F. P.; Horta, B. A. Are all-atom any better than united-atom force fields for the description of liquid properties of alkanes? *Journal of Molecular Modeling* **2020**, *26*, 1–17.

(8) Saurabh, S.; Li, Z.; Hollowell, P.; Waigh, T.; Li, P.; Webster, J.; Seddon, J. M.; Kalonia, C.; Lu, J. R.; Bresme, F. Structure and interaction of therapeutic proteins in

solution: a combined simulation and experimental study. *Molecular Physics* **2023**, *0*, e2236248.

Table S6: **Amino acid sequences adsorbing at the interface for the Gromos force-field.**

	System	Adsorbing sequences A_{ads} (amino acid) > 0.1 nm^2
1	$Gromos_{SPC}$	ARG ASP GLU LEU THR, LYS SER, LEU SER PRO GLY LYS,
		THR SER GLY GLY THR, GLY ALA LEU THR SER,
		PRO SER SER, THR GLN, ILE SER ARG THR,
		VAL GLU VAL HIS ASN, THR VAL LEU,
		PRO PRO SER ARG ASP GLU LEU THR LYS ASN GLN VAL,
		LYS SER ARG TRP GLN GLN GLY ASN VAL,
		LEU SER LEU, VAL GLY, PRO SER ARG
		LEU GLN PRO, ILE LYS ARG THR
2	$Gromos_{SPC}^{SA}$	LYS SER THR SER GLY GLY THR,
		PRO SER SER SER LEU GLY THR,
		LYS THR HIS THR,
		ARG ASP GLU LEU THR LYS ASN,
		LYS SER ARG TRP GLN,
		PRO GLY LYS,
		ALA LEU THR, THR GLN, PRO PRO,
		LEU MET ILE, GLN ASP TRP LEU ASN GLY,
		PRO PRO SER ARG ASP GLU LEU THR,
		TRP GLN GLN GLY ASN VAL ,
		HIS ASN HIS TYR THR GLN LYS SER LEU SER LEU,
		VAL GLY, GLN PRO, ARG THR VAL ALA ALA,
		GLN GLY LEU SER SER PRO VAL THR, VAL GLY,
		VAL PRO SER ARG, SER LEU GLN PRO
3	$Charmm36m_{SPCE}^{SA}$	PRO PRO SER ARG ASP GLU LEU THR LYS ASN,
		LYS SER ARG TRP GLN GLN, LEU SER PRO GLY LYS GLN VAL,
		GLY PHE THR PHE GLY SER, ILE TYR,
		SER GLY ALA LEU THR SER GLY,
		THR VAL PRO SER SER SER LEU GLY THR GLN THR TYR,
		GLN ASP TRP LEU ASN GLY,
		ALA LYS GLY GLN PRO ARG GLU PRO GLN,
		PRO PRO SER ARG ASP GLU LEU THR,
		TRP GLN GLN GLY ASN VAL, GLU ALA LEU HIS,
		GLN LYS SER LEU SER LEU SER ASP ILE GLN MET THR GLN SER PRO SER SER LEU SER ALA SER VAL,
		THR ILE THR CYS ARG ALA SER GLN SER, THR ASP PHE THR,
		LYS ARG THR VAL,
		PRO ARG GLU ALA LYS VAL GLN TRP LYS VAL ASP ASN ALA LEU,
		THR HIS GLN GLY LEU SER SER PRO VAL THR, VAL GLY,
		SER SER LEU GLN SER GLY VAL PRO SER ARG, SER SER LEU GLN PRO,
		ILE LYS ARG THR, SER LYS
